# Factors Associated with Sequelae of *Campylobacter* and Non-typhoidal *Salmonella* Infections: A Systematic Review

**DOI:** 10.1016/j.ebiom.2016.12.006

**Published:** 2016-12-08

**Authors:** Oluwaseun B. Esan, Madison Pearce, Oliver van Hecke, Nia Roberts, Dylan R.J. Collins, Mara Violato, Noel McCarthy, Rafael Perera, Thomas R. Fanshawe

**Affiliations:** a Nuffield Department of Primary Care Health Sciences, University of Oxford, United Kingdom; b National Institute for Health Research, Health Protection Research Unit, Gastrointestinal Infections, University of Oxford, United Kingdom; c Department of Zoology, University of Oxford, United Kingdom; d Bodleian Health Care libraries, University of Oxford, United Kingdom; e Health Economics Research Centre, Nuffield Department of Population Health, University of Oxford, United Kingdom; f Warwick Medical School, University of Warwick, United Kingdom

**Keywords:** Gastroenteritis, *Campylobacter*, *Salmonella*, Sequelae, Antibiotics, Acid suppression

## Abstract

Despite the significant global burden of gastroenteritis and resulting sequelae, there is limited evidence on risk factors for sequelae development. We updated and extended previous systematic reviews by assessing the role of antibiotics, proton pump inhibitors (PPI) and symptom severity in the development of sequelae following campylobacteriosis and salmonellosis. We searched four databases, including PubMed, from 1 January 2011 to 29 April 2016. Observational studies reporting sequelae of reactive arthritis (ReA), Reiter's syndrome (RS), irritable bowel syndrome (IBS) and Guillain-Barré syndrome (GBS) following gastroenteritis were included. The primary outcome was incidence of sequelae of interest amongst cases of campylobacteriosis and salmonellosis. A narrative synthesis was conducted where heterogeneity was high. Of the 55 articles included, incidence of ReA (*n* = 37), RS (*n* = 5), IBS (*n* = 12) and GBS (*n* = 9) were reported following campylobacteriosis and salmonellosis. A pooled summary for each sequela was not estimated due to high level of heterogeneity across studies (I2 > 90%). PPI usage and symptoms were sparsely reported. Three out of seven studies found a statistically significant association between antibiotics usage and development of ReA. Additional primary studies investigating risk modifying factors in sequelae of GI infections are required to enable targeted interventions.

## Highlights

• There is no clear direction of the association between antibiotics and gastroenteritis triggered reactive arthritis.
• Precision of genomic methods and increased use of record linkage techniques may provide clarity.

Antibiotics are known to change the gut flora but little is known of their potential to cause complications in patients who have gastroenteritis. We conducted a systematic review of the existing evidence to assess the potential association of antibiotic usage in patients with gastroenteritis and the occurrence of complications such as reactive arthritis (ReA). The available evidence did not indicate a clear direction in the association of antibiotics and ReA. The lack of clarity in the association of antibiotics and ReA raises a call for further primary research on the role of medications in the development of complications of gastroenteritis.

## 1 Introduction

*Campylobacter* and non-typhoidal *Salmonella enterica* (NTS) are important agents of human bacterial gastroenteritis, representing over 30% (174.3 million) of diarrhoeal illnesses globally in 2010. While *Campylobacter* was the most common cause of bacterial gastroenteritis, NTS accounted for most of the deaths caused by a bacterial foodborne agent (over 59,000) and with the highest rank for disability adjusted life years amongst foodborne disease hazards in 2010 (Havelaar et al., 2015). Globally, foodborne disease burden is not equally distributed amongst the World Health Organisation (WHO) sub regions, with the greatest burden falling on the sub regions in Africa. Nevertheless, both *Campylobacter* and NTS (henceforth gastrointestinal (GI) infections) still pose a significant disease and economic burden in developed countries (Scallan et al., 2011, Majowicz et al., 2010).

Gastroenteritis caused by *Campylobacter jejuni*/*coli* and most serotypes of NTS are characterised by a self-limiting illness without the need for medical intervention. Yet, a subset of patients develop sequelae such as reactive arthritis (ReA), Reiter's Syndrome (RS), irritable bowel syndrome (IBS), Guillain-Barré Syndrome (GBS), Inflammatory Bowel Disease (IBD), Crohn's disease (CD) and ulcerative colitis (UC) (Ajene et al., 2013, Keithlin et al., 2014, Keithlin et al., 2015).

Evidence on the factors predisposing some patients to sequelae development is limited, with only one study assessing the factors for development of IBS following enteric infection (Thabane et al., 2007). The authors found that young age, prolonged fever, anxiety and depression were risk factors for post-infectious IBS, but they did not stratify those factors by the infecting pathogen. This is a drawback for burden of disease studies, as estimates of pathogen specific sequelae development are required for prioritization of public health interventions.

In a systematic review to assess the proportion of patients who develop chronic sequelae following GI infection, the authors found that study-level factors, such as diagnosis method for complications, follow-up period from infection to sequelae development, and study size, contribute to the reported incidence of ReA and IBS following *Campylobacter* and NTS infection (Keithlin et al., 2014, Keithlin et al., 2015). However, the association of clinical factors such as proton pump inhibitors (PPI) usage and antibiotics in the development of chronic sequelae were not investigated. These drugs, which commonly increase risk of gastroenteritis, may also have a role in sequelae development due to changes to the gut microbiome and gastric pH that can favour pathogenic organisms (Doorduyn et al., 2008).

In light of the existing gap in the evidence of factors contributing to sequelae development in patients with GI infections, this systematic review extends the previous reviews to assess the study- and patient-level risk factors associated with the development of complications following *Campylobacter* and NTS infections. Specifically, we assess whether use of PPI, treatment with antibiotics and clinical symptoms such as duration of diarrhea and fever are risk factors for the development of ReA, RS, IBS, GBS, IBD, CD and UC in adults and children with a *Campylobacter* or NTS infection.

## 2 Methods

This systematic review and meta-analysis was conducted in line with the ‘Meta-analysis of Observational Studies in Epidemiology’ (MOOSE) guidelines (Stroup et al., 2000). The protocol was registered on PROSPERO (CRD 42015026042).

### 2.1 Search Strategy and Selection Criteria

We searched four electronic databases, PubMed, Agricola[http://agricola.nal.usda.gov/], EMBASE [OvidSP] (1974–2016 April 27) and CabDirect [OvidSP] (2000 to 2016 Week 15) for studies reporting sequelae of ReA, RS, IBS, GBS, IBD, CD and UC following gastrointestinal infections (*Campylobacter* and NTS). The search strategies consisted of a combination of relevant subject headings and free-text words in title and abstract for exposure and outcome. We restricted our search to studies published between 01 January 2011 and 29 April 2016 as this was an update and extension of the previous reviews with searches up to July 2011 (Keithlin et al., 2014, Keithlin et al., 2015). The detailed search strategies and results for each of the databases are presented in Supplementary Table S1. Additional eligible studies on GI infections and associated sequelae were sought by reviewing the reference lists of identified articles. No language restrictions were applied during the search.

Two reviewers independently screened titles and abstracts for relevance. Studies were included if they were cohort (prospective or retrospective), case-control, surveillance report or cross-sectional studies, or outbreak investigations of people with *Campylobacter* or NTS infection, and reported the number or proportion of people who developed the sequelae of interest following *Campylobacter* or NTS infection. Studies were excluded if they: reported sequelae without evidence of a past exposure with *Campylobacter* or NTS infection; reported sequelae with only serological evidence of past exposure to pathogens; reported sequelae for multiple foodborne infections without a breakdown of the proportion/numbers by pathogen and sequelae; were case reports, case series or experimental studies such as randomised controlled trials and laboratory based studies. We added data for the period before July 2011 from the previous systematic reviews (Keithlin et al., 2014, Keithlin et al., 2015) and extracted additional variables as required.

### 2.2 Data Extraction and Bias Assessment

Data were extracted independently by two reviewers (OE and MP) using a standardized form. Inconsistencies were resolved through a consensus process, with any disagreement resolved by a third reviewer, TF. Data coding and categorisation was in accordance with the previous reviews or otherwise stated (Supplementary Table S2) (Keithlin et al., 2014, Keithlin et al., 2015). The primary outcome was the number of cases who developed the specific sequelae of interest divided by the total number of *Campylobacter* or NTS cases.

The Joanna Briggs Institute Prevalence Critical Appraisal Tool was adapted to evaluate the quality of each study (Munn et al., 2014). This tool was selected because of its flexibility to address the risk of bias across a variety of study designs, as commonly found in the study of incidence and prevalence. Initially a calibration exercise was performed by review members using a random sample of three studies. The items in the tool were applied to the selected studies to ensure consistency across reviewers and validity in assessing the risk of bias with this tool. Following this exercise two additional questions were included (Supplementary Table S3). Inconsistency was resolved through a consensus process.

### 2.3 Statistical Analysis

We performed meta-analysis in STATA version 13 (StataCorp LP) using “metaprop_one”, a user written command for meta-analysing proportions (Nyaga et al., 2014, Freeman and Tukey, 1950). Heterogeneity was quantified using the *I*^2^ measure (Higgins et al., 2003). Where heterogeneity was high (*I*^2^ over 50%), no summary estimate was calculated.

It was possible to have multiple outcomes per study. Briefly, some studies reported multiple diagnostic methods for both pathogen and complication or multiple pathogen serotype/species. Each combination of pathogen diagnosis or sequelae diagnosis was considered as a separate outcome measure. Meta-analysis was performed using the most rigorous outcome measure based on the reference standard. For instance, where a study reported both laboratory-confirmed and probable diagnosis for a pathogen with multiple diagnostic methods for the sequelae of interest, such as self-reported diagnosis and further diagnosis by a specialist (rheumatologist for reactive arthritis), the combination of *Campylobacter*/NTS cases with a laboratory-confirmed diagnosis and the sequelae assessed by a specialist was used.

A priori subgroups to explore potential sources of heterogeneity were investigated based on relevant methodological characteristics (study design, study size, follow-up period and sequelae diagnosis) (Keithlin et al., 2014, Keithlin et al., 2015) and clinical characteristics (healthcare facility visited, symptoms of GI infections, reported PPI and antibiotic usage), if data were available.

## 3 Results

**Fig. 1:**
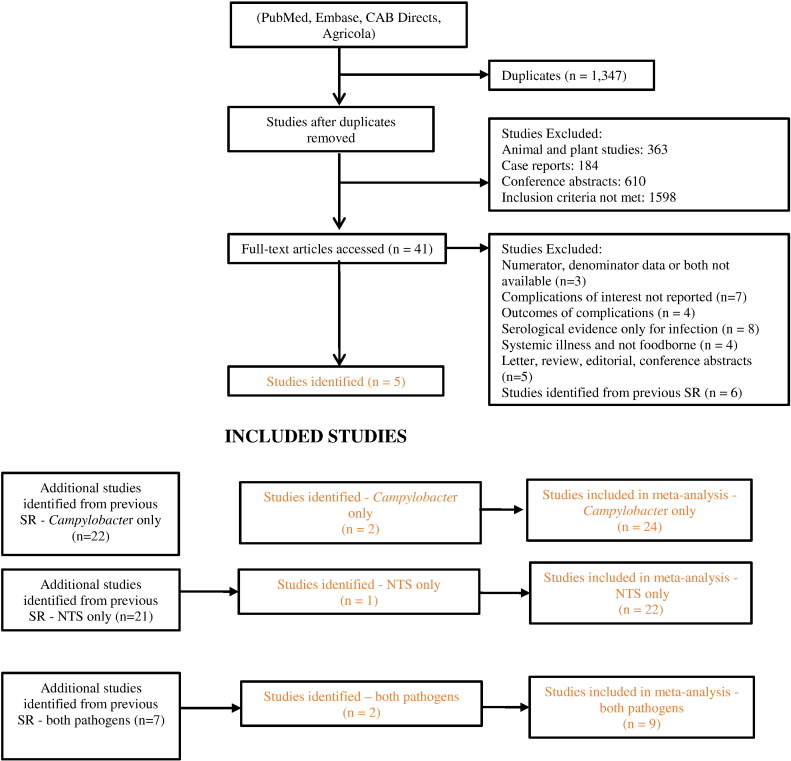
Flowchart of included studies. Results of all database searches, screening of titles and abstracts, full-text screening, additional references and selected studies.

Primary searches identified 4133 references. On removal of duplicates and after screening, five studies met the inclusion criteria reporting ReA, RS, IBS and GBS following *Campylobacter* infection (*n* = 4) and ReA, RS and IBS following NTS infection (*n* = 3) (Baker et al., 2012, Uotila et al., 2014, Porter et al., 2013a, Porter et al., 2013b, Tuompo et al., 2013). Data was extracted from all five studies and additional studies from the previous systematic reviews on ReA, RS, IBS and GBS following GI infections (*n* = 50). Hence 55 studies were included in the analysis (Fig. 1 and Table 1). No eligible studies on IBD, CD and UC were identified by our database search, so these sequelae are not considered further in this review.

Table 2 shows the characteristics of the included studies. The number of patients with GI infections varied widely (range 6 to 57,425 with *Campylobacter* and 24 to 34,664 with NTS infection) and represented all age groups. Studies reported diagnoses of the pathogen and complication according to standard practices for all cases of GI infections (87%, 48/55 and 75%, 41/55 respectively). Only 18% (10/55) reported adequate sample size calculation and, where response rate was low (49%, 27/55), only 15% (*n* = 4) adjusted for possible response bias in their analysis. An overall risk of bias score was not assigned as it was possible to have a high score (> 70%, 8.5/12) without using a reliable method of pathogen and diagnosis for all patients.

**Fig. 2:**
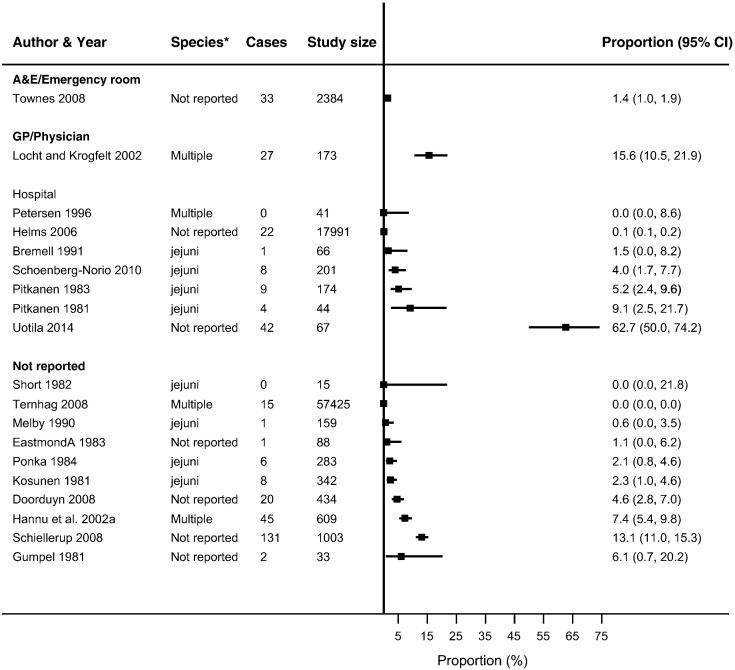
Forest plot of studies reporting incidence of *Campylobacter* triggered ReA stratified by healthcare facility. Studies reporting the incidence of reactive arthritis following *Campylobacter* infection stratified by the type of healthcare facility/practitioner visited/utilised. No summary estimate was calculated due to high heterogeneity across all studies (*I*^2^ > 90%).

**Fig. 3:**
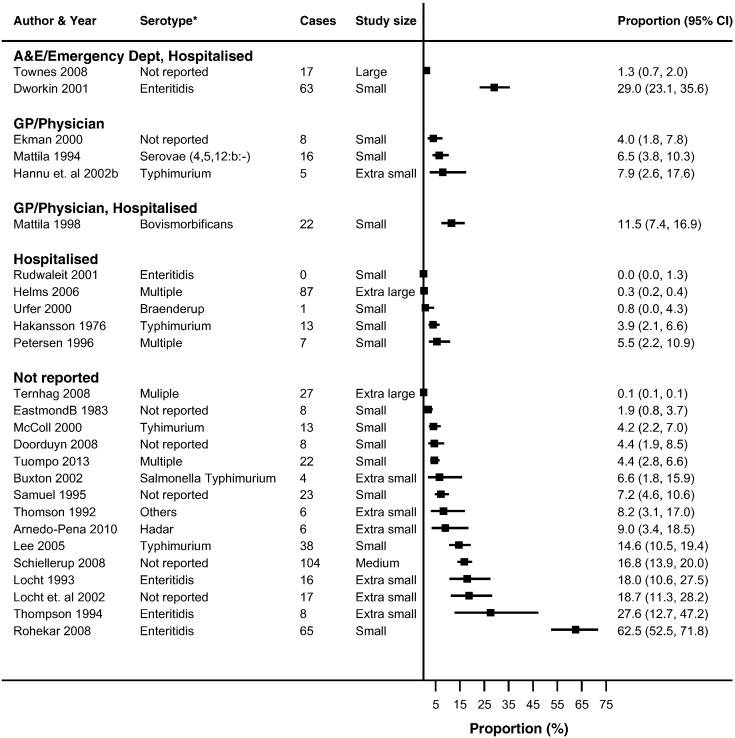
Forest plot of studies reporting incidence of NTS triggered ReA stratified by healthcare facility. Studies reporting the incidence of reactive arthritis following non-typhoidal *Salmonella* infection stratified by the type of healthcare facility/practitioner visited/utilised. No summary estimate was calculated due to high heterogeneity across all studies (*I*^2^ > 90%).

**Fig. 4:**
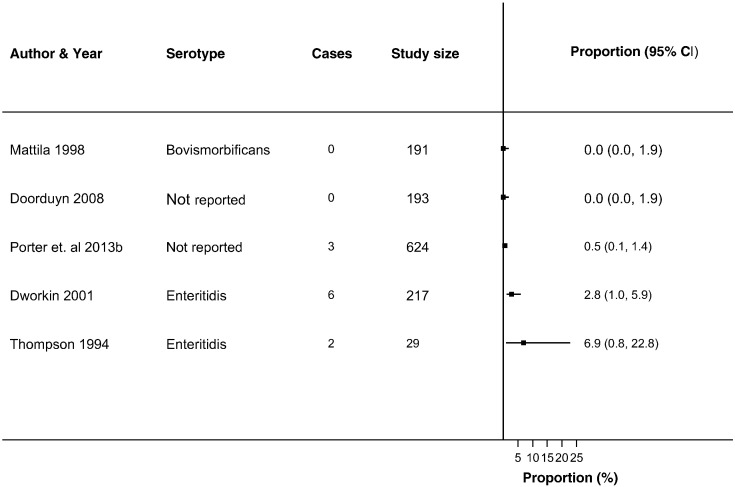
Forest plot of studies reporting incidence of RS following NTS infection. Studies reporting the incidence of Reiter's syndrome following non-typhoidal *Salmonella* infection. No summary estimate was calculated due to high heterogeneity across all studies (*I*^2^ > 90%).

**Fig. 5:**
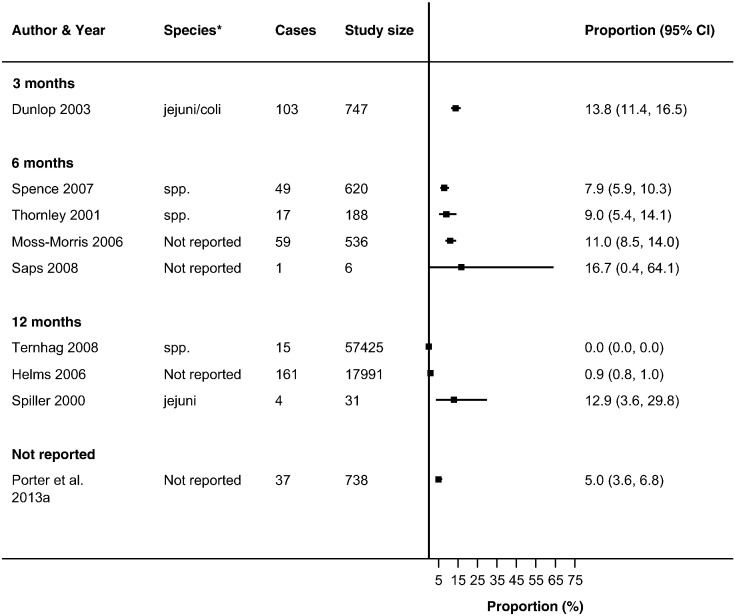
Forest plot of studies reporting incidence of IBS following *Campylobacter* infection stratified by follow-up period. Studies reporting the incidence of irritable bowel syndrome following *Campylobacter* infection stratified by the length of follow-up from infection to sequelae. No summary estimate was calculated due to high heterogeneity across all studies (*I*^2^ > 90%).

**Fig. 6:**
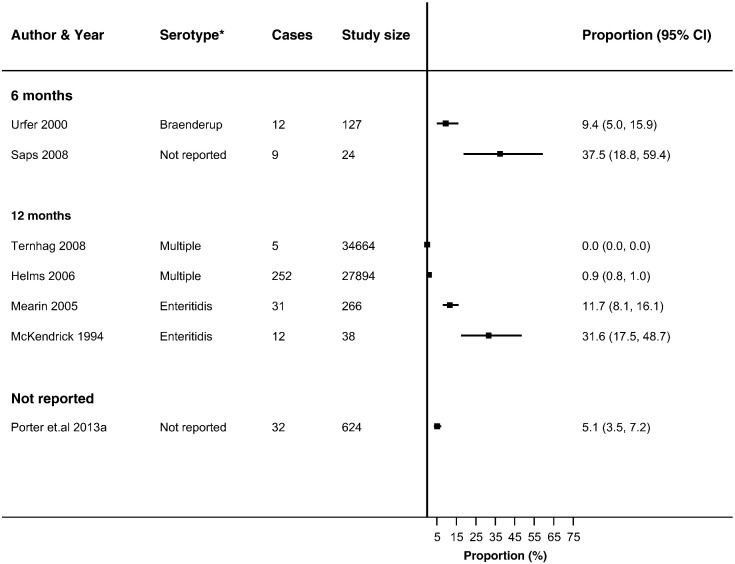
Forest plot of studies reporting incidence of IBS following NTS infection stratified by follow-up period. Studies reporting the incidence of irritable bowel syndrome following non-typhoidal *Salmonella* infection stratified by the length of follow-up from infection to sequelae. No summary estimate was calculated due to high heterogeneity across all studies (*I*^2^ > 90%).

**Fig. 7:**
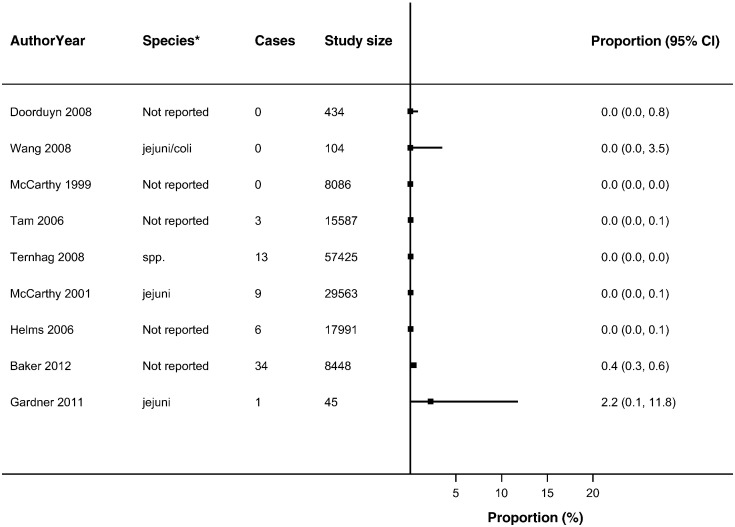
Forest plot of studies reporting incidence of GBS following *Campylobacter* infection. Studies reporting the incidence of Guillain-Barré syndrome following *Campylobacter* infection No summary estimate was calculated due to high heterogeneity across all studies (*I*^2^ > 90%).

Thirty-seven studies reported ReA following *Campylobacter* (*n* = 19) and NTS infections (*n* = 26) (Buxton et al., 2002, Bremell et al., 1991, Dworkin et al., 2001, Eastmond, 1983, Ekman et al., 2000, Gumpel et al., 1981, Hakansson et al., 1976, Hannu et al., 2002a, Hannu et al., 2002b, Helms et al., 2006, Pitkanen et al., 1981, Lee et al., 2005, Locht et al., 1993, Locht and Krogfelt, 2002, Locht et al., 2002, Mattila et al., 1994, Mattila et al., 1998, McColl et al., 2000, Petersen et al., 1996, Pitkanen et al., 1983, Ponka et al., 1984, Rohekar et al., 2008, Rudwaleit et al., 2001, Samuel et al., 1995, Schiellerup et al., 2008, Schoenberg-Norio et al., 2010, Short et al., 1982, Ternhag et al., 2008, Thomson et al., 1992, Thomson et al., 1994, Townes et al., 2008a, Tuompo et al., 2013, Uotila et al., 2014, Urfer et al., 2000, Doorduyn et al., 2008, Arnedo-Pena et al., 2010, Eastmond et al., 1983, Melby et al., 1990) in up to 63% of patients with either infection (Supplementary Table 4). The majority of studies reported ReA triggered by *Campylobacter* (*n* = 14) or NTS infection (*n* = 18) in < 10% of patients with gastroenteritis. No overall summary of incidence of sequelae following gastroenteritis were calculated, as there was a high level of heterogeneity across studies (*I*^2^ > 90%) (Fig. 2, Fig. 3, Fig. 4, Fig. 5, Fig. 6, Fig. 7). The incidences of RS, IBS and GBS were reported in 5, 12, and 9 studies respectively (Fig. 4, Fig. 5, Fig. 6, Fig. 7 and Table S4). < 10% of patients with either infection developed RS in all studies. Incidence of IBS was reported in *Campylobacter* (0% to 18%) and NTS (0% to 38%) patients. GBS was less frequent with all but one study reporting an incidence of < 2% following *Campylobacter* infection.

Only studies reporting incidence of ReA considered the use of PPI and antibiotics as potential factors contributing to sequelae development. One study assessing the use of PPI in the development of ReA following *Campylobacter* and NTS infection found a significant association after adjustment for age, sex and degree of urbanization (adjusted OR 2.9 (95% CI 1.4–6.1)) (Doorduyn et al., 2008).

The prescription/usage of antibiotics was reported in 17 studies (*n* = 7 for *Campylobacter* and *n* = 11 for NTS) (Arnedo-Pena et al., 2010, Buxton et al., 2002, Rudwaleit et al., 2001, Ekman et al., 2000, Schonberg-Norio et al., 2010, Dworkin et al., 2001, Mattila et al., 1998, Hannu et al., 2002b, Townes et al., 2008b, Tuompo et al., 2013, Lee et al., 2005, Pitkanen et al., 1981, Pitkanen et al., 1983, Ponka et al., 1984, Locht and Krogfelt, 2002, Locht et al., 1993, Uotila et al., 2014). Seven studies considered the risk of ReA development following antibiotics usage (Arnedo-Pena et al., 2010, Buxton et al., 2002, Dworkin et al., 2001, Mattila et al., 1998, Hannu et al., 2002b, Locht and Krogfelt, 2002, Townes et al., 2008b). Of these studies, increased risk of ReA development was observed following treatment of infection with antibiotics (class not provided) for *Campylobacter* and fluoroquinolones for NTS infection (*n* = 1 and 2, respectively) (Locht and Krogfelt, 2002, Dworkin et al., 2001, Mattila et al., 1998). Three studies found a protective effect in the use of antibiotics for NTS; however the findings were not statistically significant (Arnedo-Pena et al., 2010, Buxton et al., 2002, Hannu et al., 2002b). The remaining study did not find any associated risk with antibiotic usage for either *Campylobacter* or NTS infection. Moreover the data were not reported in the study (Townes et al., 2008b) (Table 3). Studies assessing association of antibiotic treatment and development of ReA reported visit to a GP/Physician (*n* = 4) or accident and emergency/hospital (*n* = 3).

Clinical symptoms were scarcely reported by studies and, where available, studies used different definitions and thresholds for diarrhea and fever (data not shown). This prevented further analysis of the role of symptom severity in sequelae development.

For the methodological subgroup analysis, only consultation with a rheumatologist statistically reduced heterogeneity of studies within the “specialist” in *Campylobacter* and NTS triggered ReA (Table 4A, Table 4B, Table 4C, Table 4D, Fig. 2, Fig. 3). Follow-up may have contributed to heterogeneity in *Campylobacter* triggered IBS as studies within the 6-month follow-up stratum had a statistically significant reduction in heterogeneity (*I*^2^ = 24.2%) (Table 4A, Table 4B, Table 4C, Table 4D, Fig. 6). The only clinical characteristic considered in a subgroup analysis was the healthcare facility visited for the GI infection in those developing ReA and IBS. Studies reporting a “GP/Physician” visit for NTS infection were fairly homogenous (*I*^2^ = 2.0%), nonetheless high heterogeneity remained in this stratum for patients with *Campylobacter* triggered ReA (Fig. 2, Fig. 3).

## 4 Discussion

Previous systematic reviews considering incidence of ReA, RS, IBS and GBS conducted literature searches up until 2011 without assessing factors contributing to sequelae development. Five years after these searches were conducted, we only identified five new studies based on our inclusion criteria. We found that use of PPI and antibiotics may be possible factors associated with the development of ReA following GI infections. These factors were sparsely reported by studies and where information was available high heterogeneity (*I*^2^ > 90%) prevented the pooling of data.

Despite the over prescription of PPI in both primary and secondary settings (Forgacs and Loganayagam, 2008), only one study reported an association of PPI usage and ReA development in patients with gastroenteritis (Doorduyn et al., 2008). The authors demonstrated that PPI usage was independent of a single nucleotide polymorphism (SNP) in interferon gamma (IFN-γ) in cases with *Campylobacter* and NTS infections and the development of reactive arthritis (Doorduyn et al., 2008). IFN-γ is a cytokine crucial in the immune response against enteric infections. The combination of a SNP in IFN-γ with PPI usage could lead to increased susceptibility to enteric infections and subsequent prolonged or repeated episodes of GI infection. These sequential events may increase the susceptibility to reactive arthritis (Doorduyn et al., 2008).

Of all the sequelae considered, only risk of reactive arthritis was assessed following antibiotic usage in cases of *Campylobacter* and NTS infection. The associated risk is not clear as the studies report elevated, decreased or no risk of ReA following GI infection. The association may be dependent on the dose and type of antibiotics, as evidenced in a systematic review (Agger et al., 2015) evaluating the risk of hemolytic uremic syndrome following the use of antibiotics in patients with shiga toxin producing *Escherichia coli* (STEC) infections. Agger et al. (2015) showed that protein and cell wall synthesis class of antibiotics may be protective and improve recovery time and proposed a review of guidelines on contraindication of antibiotics for STEC infections.

Most gastroenteritis cases do not require treatment with antibiotics unless they are severe and occur in at risk groups i.e. elderly, children and those with underlying comorbidities. Due to insufficient information in the studies, we could not assess the potential reason for antibiotic treatment or the risk associated with sequelae development following antibiotic usage in a meta-analysis. Moreover, information on duration of treatment, dose of antibiotics, age and gender of all cases who received antimicrobial treatment were not available in all of the studies, thereby limiting any further comparisons or pooling of data.

Heterogeneity could not be explained by most of the subgroup analysis considered, except in the reported method of diagnosing reactive arthritis complication following *Campylobacter* and NTS infection, the type of healthcare facility visited for an NTS infection and follow-up period in *Campylobacter* triggered IBS. In studies reporting a visit to the GP/Physicians in cases of NTS infection the heterogeneity was significantly reduced (*I*^2^ = 2%). The three studies were all conducted in Finland, reported specialist diagnosis for reactive arthritis, had laboratory confirmation of the NTS infection and two assessed use of antibiotics in development of ReA in NTS patients. A GP/physician consultation may lead to a laboratory confirmed diagnosis of infection, prescription of antibiotics, and referral to a specialist; hence influencing the reported sequelae incidence. The small number of studies with inconsistent follow-up period from NTS infection to ReA development limits further interpretation of this finding.

Follow-up period may be crucial in the reported incidence of IBS following campylobacteriosis, due to a statistically significant reduction in heterogeneity in studies reporting a 6-month follow-up period, despite using different versions of Rome I, II and III criteria for diagnosis. The current gold-standard for IBS diagnosis is Rome III classification, which is symptom-based, requiring patients to be symptomatic both at 3 and 6 months after initial symptom onset (Longstreth et al., 2006) was used by only one study (Spence and Moss-Morris, 2007). Potential risk modifying factors in the development of IBS following gastroenteritis were not evaluated due to insufficient reporting and primary aim of included studies.

Data source may be a potential source of unexplained heterogeneity even though it was not significant in our subgroup analysis (data not shown). Using a combination of studies reporting outbreaks, population surveillance and hospital surveillance may have introduced additional heterogeneity. Outbreaks are usually caused by a single strain, while population surveillance identifies the circulating strains. In one of the studies included in this review, Tuompo et al. (2013) used phenotypic methods to determine potential differences in the O antigens of different NTS serotypes circulating in a population surveillance. However no significant differences in the arthritogenicity of the serotypes to trigger ReA were identified (Tuompo et al., 2013). The precision of genomic methods, such as whole genome sequencing, may provide further insights into differing potential for bacterial strains to trigger sequelae.

Our study has a number of major strengths. Firstly, our study addresses an existing knowledge gap by assessing the available evidence on factors contributing to the development of sequelae following *Campylobacter* and NTS infection. This is important in understanding factors amenable to intervention. Secondly, our review highlights the need for investigators to consider remediable risk factors in sequelae development following GI infections.

The reliance of observational studies on “natural experiments” such as outbreaks makes them prone to low study quality and high level of bias and heterogeneity. Most outbreak investigations are set up as a rapid response to an emergency situation to determine the cause of the outbreak. Time constraints limits the opportunity to address secondary factors that may improve the overall evidence on GI triggered sequelae. Therefore, it was possible for a study to have a high quality score for the primary research but missing key items such as method of diagnosis of pathogen and sequelae, crucial for risk factor study. Thus, a quality criteria was not adopted for inclusion of studies for meta-analysis. Although heterogeneity was significantly reduced for studies reporting a specialist consultation for diagnosis of ReA following either *Campylobacter* or NTS infection, we did not report a pooled estimate. Specialist diagnosis was preceded by self-reported symptoms. Not all patients with symptoms were seen by a specialist. Further, the studies reported different follow-up times indicating different definitions of ReA were adopted.

Other limitations of our study include inconsistencies in the reporting of key features, such as age, definition of sequelae, and follow-up of gastroenteritis cases in included studies. The risk factors we considered were also sparsely reported. Further, unexplained sources of heterogeneity prevented the reporting of a summary statistic for factors contributing to sequelae development. These limit the application of estimates to the general population and made interpretation of results difficult.

In conclusion, the findings of this systematic review show that the factors contributing to sequelae development following GI infections remain unclear. This is a challenge for policy makers in targeting interventions to reduce the overall burden of GI infections. Researchers should consider reporting information that will improve the overall evidence of sequelae of GI infections when observational studies are conducted. Applying record linkage in following up patients' health journey to study sequelae of GI infections may improve reporting of observational studies.

Furthermore, primary research in risk modifiers of gastroenteritis triggered sequelae and the potential for different bacterial strains to cause sequelae may address the knowledge gap in the relationship between pathogen and host.

## Conflict of Interest

Prof. Perera received grants from UK NIHR, during the conduct of the study.

## Author Contributions

OE wrote the manuscript. OE conceived the initial idea for the study, NM, MV, RP and TF critically appraised the protocol, manuscript and also contributed to its development by revising different versions. NR assisted with the database searches and ran the updated searches. OE and OvH screened all abstracts; OE and DC completed the full text screening; OE and MP performed all data extraction and risk of bias was completed by OE, MP and TF. All authors approved the final version and take responsibility for its content.

## Figures and Tables

**Table 1:** Included studies by pathogen and sequelae reported.

Sequelae	*Campylobacter*	NTS	*Campylobacter* & NTS
Reactive arthritis	Gumpel et al. (1981), Kosunen et al. (1981), Pitkanen et al. (1981), Short et al. (1982), Eastmond et al. (1983), Pitkanen et al. (1983), Ponka et al. (1984), Melby et al. (1990), Bremell et al. (1991), Hannu et al. (2002a), Locht and Krogfelt (2002), Schoenberg-Norio et al. (2010) and Uotila et al. (2014)	Hakansson et al. (1976), Eastmond (1983), Thomson et al. (1992), Locht et al. (1993), Mattila et al. (1994), Thomson et al. (1994), Samuel et al. (1995), Mattila et al. (1998), Ekman et al. (2000), McColl et al. (2000), Urfer et al. (2000), Dworkin et al. (2001), Rudwaleit et al. (2001), Buxton et al. (2002), Hannu et al. (2002b), Locht et al. (2002), Lee et al. (2005), Rohekar et al. (2008), Arnedo-Pena et al. (2010) and Tuompo et al. (2013)	Petersen et al. (1996), Helms et al. (2006), Doorduyn et al. (2008), Schiellerup et al. (2008), Ternhag et al. (2008) and Townes et al. (2008b)
Reiter's syndrome	None	Thomson et al. (1994), Mattila et al. (1998) and Dworkin et al. (2001)	Doorduyn et al. (2008) and Porter et al. (2013b)
Irritable bowel syndrome	Spiller et al. (2000), Thornley et al. (2001), Dunlop et al. (2003), Moss-Morris and Spence (2006), and Spence and Moss-Morris (2007)	McKendrick and Read (1994), Urfer et al. (2000) and Mearin et al. (2005)	Helms et al. (2006), Saps et al. (2008), Ternhag et al. (2008), and Porter et al. (2013a)
Guillain Barré Syndrome	McCarthy et al. (1999), McCarthy and Giesecke (2001), Helms et al. (2006), Tam et al. (2006), Ternhag et al. (2008), Doorduyn et al. (2008), Wang et al. (2008), Gardner et al. (2011) and Baker et al. (2012)	None	None

**Table 2:** Study characteristics for complications following *Campylobacter* and non-typhoidal *Salmonella* infection.

First author, year	Country	Study design	Data source	Outbreak source	Date_Data collection	Season	Age	% Female	Complication
*Campylobacter* only									
Baker et al. (2012)	New Zealand	Prospective population surveillance	Hospital records	N.A.	1995–2008	All	All ages	N.R.	GBS
Bremell et al. (1991)	Sweden	Prospective outbreak	Outbreak in community	Unknown	1981	Autumn	Adults	56%	ReA
Dunlop et al. (2003)	England	Prospective population surveillance	Surveillance of population (sporadic or outbreak)	N.A.	1999–2002	Various	Adult	N.R.	IBS
Eastmond et al. (1983)	Scotland	Retrospective outbreak	Outbreak in community	Food - dairy	1979	Winter	N.R.	N.R.	ReA
Gardner et al. (2011)	Canada	Prospective outbreak	Outbreak in community	Food - vegetable	2008	Autumn	All ages	51%	GBS
Gumpel et al. (1981)	England	Retrospective population surveillance	Surveillance of population (sporadic or outbreak)	N.A.	1978	All	All ages	N.R.	ReA
Hannu et al. (2002a)	Finland	Prospective population surveillance	Surveillance of population (sporadic or outbreak)	N.A.	1997–1998	All	All ages	59%	ReA
Kosunen et al. (1981)	Finland	Unknown	Surveillance of population (sporadic or outbreak)	N.A.	1978–1979	All	N.R.	N.R.	ReA
Locht and Krogfelt (2002)	Denmark	Retrospective population surveillance	Surveillance of population (sporadic or outbreak)	N.A.	1997–2000	All	Adults	57%	ReA
McCarthy et al. (1999)	Sweden	Retrospective outbreak	Outbreak in community	Waterborne	1980, 1994, 1995	Various	N.R.	N.R.	GBS
McCarthy and Giesecke (2001)	Sweden	Retrospective population surveillance	Disease registry	N.A.	1987–1995	All	All ages	N.R.	GBS
Melby et al. (1990)	Norway	Retrospective outbreak	Outbreak in community	Waterborne	Pre-1990	Spring/summer	All ages	48%	ReA
Moss-Morris and Spence (2006)	New Zealand	Prospective population surveillance	Surveillance of population (sporadic or outbreak)	N.A.	2002–2003	Various	Adults	N.R.	IBS
Pitkanen et al. (1983)	Finland	Prospective hospital surveillance	Hospital records	N.A.	1978–1981	All	All ages	47%	ReA
Pitkanen et al. (1981)	Finland	Prospective hospital surveillance	Hospital records	N.A.	1978–1980	All	All ages	46%	ReA
Ponka et al. (1984)	Finland	Prospective population surveillance	Surveillance of population (sporadic or outbreak)	N.A.	1978–1981	All	N.R.	N.R.	ReA
Schoenberg-Norio et al. (2010)	Finland	Cross sectional	Surveillance of population (sporadic or outbreak)	N.A.	2002	Summer	All ages	48%	ReA
Short et al. (1982)	UK	Prospective	Hospital records	N.A.	1979	Various	N.R.	N.R.	ReA
Spence and Moss-Morris (2007)	New Zealand	Prospective	Surveillance of population (sporadic or outbreak)	N.A.	Pre-2006	All	Adults	N.R.	IBS
Spiller et al. (2000)	UK	Prospective population surveillance	Hospital records	N.A.	Pre-2000	All	Adult	N.R.	IBS
Tam et al. (2006)	UK	Retrospective population surveillance	Disease registry	N.A.	1991–2001	All	N.R.	N.R.	GBS
Thornley et al. (2001)	UK	Prospective population surveillance	Surveillance of population (sporadic or outbreak)	N.A.	1997	Spring/summer	Adults	N.R.	IBS
Uotila et al. (2014)	Finland	Retrospective outbreak	Outbreak in community	Waterborne	2007	Winter	All ages	73%	ReA
Wang et al. (2008)	China	Retrospective population surveillance	Hospital records	N.A.	2000–2006	All	Children	30%	GBS

NTS only									
Arnedo-Pena et al. (2010)	Spain	Prospective outbreak	Outbreak in community	Food - meat	2005	Summer	All ages	49.7%	ReA
Buxton et al. (2002)	Canada	Prospective population surveillance	Surveillance of population (sporadic or outbreak)	N.A.	1999–2000	All	All ages	53.0%	ReA
Dworkin et al. (2001)	USA	Prospective outbreak	Outbreak in community	Food - meat	1994	Autumn/winter	Adults	58.5%	ReA, Reiter's
Eastmond (1983)	Scotland	Prospective outbreak	Outbreak in community	Food - dairy	1981	Autumn	All ages	47.8%	ReA
Ekman et al. (2000)	Finland	Prospective population surveillance	Surveillance of population (sporadic or outbreak)	N.A.	1998–1999	All	NR	53.0%	ReA
Hakansson et al. (1976)	Sweden	Retrospective outbreak	Outbreak in community		1974	N.R.	Adults	N.R.	ReA
Hannu et al. (2002b)	Finland	Prospective outbreak	Outbreak in community	Unknown	1999	Spring/summer	Adults & Children	56.9%	ReA
Lee et al. (2005)	Australia	Retrospective outbreak	Outbreak in community	Food - vegetable	1999	Various	All ages	48.3%	ReA
Locht et al. (1993)	Finland	Retrospective outbreak	Outbreak in community	Food - other	1990	Spring	Adults	44.4%	ReA
Locht et al. (2002)	Denmark	Prospective outbreak	Outbreak in community	Food - other	1999	Winter	Adults	56.0%	ReA
Mattila et al. (1994)	Finland	Prospective outbreak	Outbreak in community	Food - vegetable	1992	Autumn	All ages	62.2%	ReA
Mattila et al. (1998)	Finland	Prospective outbreak	Outbreak in community	Food - vegetable	1994	Spring	All ages	68.1%	ReA & Reiter's
McColl et al. (2000)	Australia	Prospective outbreak	Outbreak in community	Food - meat	1997	Spring	All ages	51.0%	ReA
McKendrick and Read (1994)	UK	Prospective outbreak	Outbreak in community	Food- other	Pre-1994	N.R.	N.R.	65.8%	IBS
Mearin et al. (2005)	Spain	Prospective outbreak	Outbreak in community	Food - dairy	2002	Summer	Adults	55.3%	IBS
Rohekar et al. (2008)	Canada	Retrospective outbreak	Outbreak in community	Food - vegetable	2005	Autumn/winter	Adults	71.2%	ReA
Rudwaleit et al. (2001)	Germany	Prospective outbreak	Outbreak in community	Food - dairy	1998	Winter	Children	N.R.	ReA
Samuel et al. (1995)	USA	Retrospective outbreak	Outbreak in community	Unknown	1993	Summer	NR	N.R.	ReA
Thomson et al. (1994)	Canada	Retrospective outbreak	Outbreak in community	Food - meat	1990	Spring	NR	N.R.	ReA
Thomson et al. (1992)	Canada	Prospective outbreak	Outbreak in community	Food - meat	Pre-1992	N.R.	Adults	94.5%	ReA
Tuompo et al. (2013)	Finland	Prospective population surveillance	Surveillance of population (sporadic or outbreak)	NA	2003–2004	Various	All ages	60.1%	ReA
Urfer et al. (2000)	Switzerland	Prospective outbreak	Outbreak in community	Food - meat	1993	Autumn	All ages	37.2%	IBS & ReA

*Campylobacter* and NTS									
Doorduyn et al. (2008)	Netherlands	Prospective population surveillance	Surveillance of population (sporadic or outbreak)	N.A.	2002–2003, 2005	All	NR	N.R.	GBS, ReA, Reiter's
Helms et al. (2006)	Denmark	Retrospective population surveillance	Disease registry	N.A.	1991–1999	All	All ages	52%	GBS, IBS, ReA
Petersen et al. (1996)	Denmark	Retrospective hospital surveillance	Hospital records		1991–1993	All	All ages	N.R.	ReA
Porter et al. (2013b)	USA	Retrospective population surveillance	Disease registry	N.A.	1998 to 2009	All	Adults	N.R.	Reiter's
Porter et al. (2013a)	USA	Retrospective population surveillance	Disease registry	N.A.	1998 to 2009	All	Adults	N.R.	IBS
Saps et al. (2008)	USA & Italy	Prospective population surveillance	Hospital records	N.A.	2006	Various	Children	N.R.	IBS
Schiellerup et al. (2008)	Denmark	Prospective population surveillance	Surveillance of population (sporadic or outbreak)	N.A.	2002–2003	All	Adults	57%	ReA
Ternhag et al. (2008)	Sweden	Retrospective population surveillance	Surveillance of population (sporadic or outbreak)	N.A.	1997–2004	All	All ages	*Campylobacter* - 47% NTS - 50.6%	GBS, IBS, ReA
Townes et al. (2008b)	USA	Prospective population surveillance	Surveillance of population (sporadic or outbreak)	N.A.	2002–2004	All	All ages	*Campylobacter* - 46.9% NTS - 56.0%	ReA

N.A. – not applicable; N.R. – not reported.

**Table 3:** Risk of developing ReA following prescription/usage of antibiotics in Campylobacter and NTS patients stratified by healthcare facility accessed.

Author_year	Pathogen	No. with pathogen	Antibiotics usage N (%)	Antibiotics	Proportion of ReA vs. non-ReA using antibiotics
Visited general practitioner/physician					
Locht and Krogfelt (2002)	*Campylobacter*	173	56 (32)	N.R.	56% with ReA vs. 26% non-ReA; *p* = 0.03^a^, ^d^
Arnedo-Pena et al. (2010)	*S*. Hadar PT2	155	57 (38)^b^	Fluoroquinolones	aRR^c^ 0.43; 95% CI 0.17–1.08
Hannu et al. (2002b)	*S*. Typhimurium DT193	63	32 (63)^b^	Fluoroquinolones	0% with ReA vs. 16% no ReA; *p* = 0.056
Mattila et al. (1998)	*S*. Bovismorbificans	191	78 (41)	Fluoroquinolones	59% with ReA vs. 35% non-ReA; *p* = 0.021^a^, ^d^

Visited accident & emergency/hospitalised					
Dworkin et al. (2001)	*S*. Enteritidis	217	66 (30)	Fluoroquinolones	RR 1.6; 95% CI 1.1–2.5^a^
Townes et al. (2008b)	NTS	1356	365 (27)	Quinolone, β-lactam and macrolide	No associated risk (data not reported)
Townes et al. (2008b)	*Campylobacter*	2384	1978 (83)	Quinolone, β-lactam and macrolide	No associated risk (data not reported)

Visit of healthcare facility not reported					
Buxton et al. (2002)	*S*. Typhimurium	61	28 (46)	N.R.	OR 0.29; 95% CI 0.07–1.18

a Antibiotics information not available for all with gastroenteritis.

b Adjusted relative risk.

c Significant results.

d Chi-squared test in comparison of proportion.

**Table 4A:** Subgroup meta-analysis for studies reporting development of reactive arthritis following *Campylobacter* infection by sequelae diagnosis, follow-up period, study size and healthcare facility visited.

Variable	*I*^2^	Number of studies
*Sequelae diagnosis*		
Physician/medical records	95.6%	6
Self-reported disease status	97.0%	6
Self-reported disease status based on a validated scale	–	1
Specialist^a^	48.6%	2
Combination	0.0%	3
Not reported	–	1

*Follow-up period*		
< 3 months	97.0%	8
3 months	–	1
> 3 months < 1 year	–	–
1 year	95.6%	3
> 1 year	97.8%	2
Not reported	56.4%	5

*Study size*		
Extra small (*n* < 100)	95.3%	7
Small (101–500)	86.3%	7
Medium (501–1000)	–	1
Large (1001–10,000)	97.70%	2
Extra-large (> 10.000)	99.20%	2

*Healthcare facility visited*		
GP/physician	–	1
GP/hospitalised	–	–
A&E/hospitalised	–	1
Hospitalised	97.8%	7
Not reported	98.8%	10

a Heterogeneity significantly reduced (*I*^2^ < 50%).

**Table 4B:** Subgroup meta-analysis for studies reporting development of reactive arthritis following NTS infection by sequelae diagnosis, follow-up period, study size and healthcare facility visited.

Variable	*I*^2^	Number of studies
*Sequelae diagnosis*		
Physician/medical records	97.7%	9
Self-reported disease status	96.8%	9
Self-reported disease status based on a validated scale	–	1
Specialist^a^	41.2%	6
Combination	–	1
Not reported	–	1

*Follow-up period*		
< 3 months	97.3%	10
3 months	81.3%	5
> 3 months < 1 year	93.5%	4
1 year	97.8%	2
> 1 year	95.3%	2
Not reported	97.7%	4

*Study size*		
Extra small (*n* < 100)	58.8%	7
Small (101–500)	95.6%	16
Medium (501–1000)	96.1%	1
Large (1001–10,000)	–	1
Extra-large (> 10.000)	99.80%	1

*Healthcare facility visited*		
GP/physician^a^	2.0%	3
GP/hospitalised	–	1
A&E/hospitalised	99.9%	2
Hospitalised	91.7%	5
Not reported	98.8%	20

a Heterogeneity significantly reduced (*I*^2^ < 50%).

**Table 4C:** Subgroup meta-analysis for studies reporting development of irritable bowel syndrome following *Campylobacter* infection by sequelae diagnosis, follow-up period, study size and healthcare facility visited.

Variable	*I*^2^	Number of studies
*Sequelae diagnosis*		
Physician/medical records	99.6%	3
Self-reported disease status based on a validated scale	63.2%	6

*Follow-up period*		
3 months	–	1
> 3 months < 1 year⁎	24.2%	4
1 year	99.4%	3
Not reported	–	1

*Study size*		
Extra small (*n* < 100)	87.7%	3
Small (101–500)	–	1
Medium (501–1000)	92.2%	4
Extra large (> 10.000)	99.90%	2

⁎ Heterogeneity significantly reduced (*I^2^ < 50%*).

**Table 4D:** Subgroup meta-analysis for studies reporting development of irritable bowel syndrome following NTS infection by sequelae diagnosis, follow-up period, study size and healthcare facility visited.

Variable	*I*^2^	Number of studies
*Sequelae diagnosis*		
Physician/medical records	99.6%	3
Self-reported disease status	–	1
Self-reported disease status based on a validated scale	87.3%	3

*Follow-up period*		
> 3 months < 1 year	99.8%	2
1 year	99.5%	4
Not reported	–	1

*Study size*		
Extra small (*n* < 100)	87.7%	3
Small (101–500)	–	1
Medium (501–1000)	–	1
Extra-large (> 10.000)	99.20%	2

*Healthcare facility*		
Hospitalised	99.70%	2
Not reported	98.8%	5

## Appendix A Supplementary data

Supplementary material Image 1
