# Supplementary material for: Factors Associated with Sequelae of Campylobacter and Non-typhoidal Salmonella Infections: A Systematic Review
Source: eBioMedicine. 2016 Dec 8;15:100–11. doi: 10.1016/j.ebiom.2016.12.006 (PMC5233817; doi:10.1016/j.ebiom.2016.12.006)
Supplement: Supplementary file 1 — Supplementary material [file mmc1.docx]

**SUPPLEMENTARY MATERIALS**

**Table of Contents**

[Table 1: Searches 2](#_Toc463467270)

[Pubmed 2](#_Toc463467271)

[Embase 3](#_Toc463467272)

[CAB Abstracts 4](#_Toc463467273)

[Agricola 5](#_Toc463467274)

[Search summary 5](#_Toc463467275)

[Table 2. Data extraction tool 6](#_Toc463467276)

[Table 3. Risk of Bias Tool 13](#_Toc463467277)

[Table 4A. Proportion developing complications following *Campylobacter* infection 16](#_Toc463467278)

[Table 4B. Proportion developing complications following NTS infection 19](#_Toc463467279)

#

# Supplementary Table S1: Searches

## Pubmed

| **Search** | **Query** | **Items found** |
| --- | --- | --- |
| #25 | Search (((("Campylobacter Infections"[Mesh]) OR "Salmonella Infections"[Mesh]) OR ((campylobacter*[Title/Abstract]) OR salmonella*[Title/Abstract]))) AND (((((((#9) OR "Guillain-Barre Syndrome"[Mesh]) OR "Inflammatory Bowel Diseases"[Mesh]) OR "Irritable Bowel Syndrome"[Mesh]) OR "Arthritis, Reactive"[Mesh]) OR "Hemolytic-Uremic Syndrome"[Mesh]) OR (Sequel* OR Complication* OR Long-term OR Longterm OR chronic OR Guillain* OR HUS OR "Haemolytic uremic syndrome" OR "Hemolytic uremic syndrome" OR "Hemorrhagic uremic syndrome" OR "Haemorrhagic uraemic syndrome" OR Reiter* OR Arthritis* OR "Irritable bowel syndrome" OR IBS OR "inflammatory bowel disease" OR colitis OR crohn*)) Filters: Publication date from 2011/01/01 to 2016/12/31 | 1536 |
| #24 | Search (((("Campylobacter Infections"[Mesh]) OR "Salmonella Infections"[Mesh]) OR ((campylobacter*[Title/Abstract]) OR salmonella*[Title/Abstract]))) AND (((((((#9) OR "Guillain-Barre Syndrome"[Mesh]) OR "Inflammatory Bowel Diseases"[Mesh]) OR "Irritable Bowel Syndrome"[Mesh]) OR "Arthritis, Reactive"[Mesh]) OR "Hemolytic-Uremic Syndrome"[Mesh]) OR (Sequel* OR Complication* OR Long-term OR Longterm OR chronic OR Guillain* OR HUS OR "Haemolytic uremic syndrome" OR "Hemolytic uremic syndrome" OR "Hemorrhagic uremic syndrome" OR "Haemorrhagic uraemic syndrome" OR Reiter* OR Arthritis* OR "Irritable bowel syndrome" OR IBS OR "inflammatory bowel disease" OR colitis OR crohn*)) | 11047 |
| #23 | Search ((((((#9) OR "Guillain-Barre Syndrome"[Mesh]) OR "Inflammatory Bowel Diseases"[Mesh]) OR "Irritable Bowel Syndrome"[Mesh]) OR "Arthritis, Reactive"[Mesh]) OR "Hemolytic-Uremic Syndrome"[Mesh]) OR (Sequel* OR Complication* OR Long-term OR Longterm OR chronic OR Guillain* OR HUS OR "Haemolytic uremic syndrome" OR "Hemolytic uremic syndrome" OR "Hemorrhagic uremic syndrome" OR "Haemorrhagic uraemic syndrome" OR Reiter* OR Arthritis* OR "Irritable bowel syndrome" OR IBS OR "inflammatory bowel disease" OR colitis OR crohn*) | 4189495 |
| #22 | Search Sequel* OR Complication* OR Long-term OR Longterm OR chronic OR Guillain* OR HUS OR "Haemolytic uremic syndrome" OR "Hemolytic uremic syndrome" OR "Hemorrhagic uremic syndrome" OR "Haemorrhagic uraemic syndrome" OR Reiter* OR Arthritis* OR "Irritable bowel syndrome" OR IBS OR "inflammatory bowel disease" OR colitis OR crohn* | 4079420 |
| #20 | Search "Hemolytic-Uremic Syndrome"[Mesh] | 4978 |
| #18 | Search "Arthritis, Reactive"[Mesh] | 3802 |
| #16 | Search "Irritable Bowel Syndrome"[Mesh] | 5141 |
| #14 | Search "Inflammatory Bowel Diseases"[Mesh] | 65279 |
| #12 | Search "Guillain-Barre Syndrome"[Mesh] | 3892 |
| #9 | Search "complications" [Subheading] Schema: syn | 1825334 |
| #7 | Search (("Campylobacter Infections"[Mesh]) OR "Salmonella Infections"[Mesh]) OR ((campylobacter*[Title/Abstract]) OR salmonella*[Title/Abstract]) | 90649 |
| #6 | Search (campylobacter*[Title/Abstract]) OR salmonella*[Title/Abstract] | 77934 |
| #5 | Search "Salmonella Infections"[Mesh] | 32354 |
| #2 | Search "Campylobacter Infections"[Mesh] | 6381 |

##

## Embase

| **# ▲** | **Searches** | **Results** |
| --- | --- | --- |
| 1 | (salmonella* or campylobacter*).ti,ab. | 82925 |
| 2 | exp salmonellosis/ | 27049 |
| 3 | campylobacteriosis/ | 1205 |
| 4 | 1 or 2 or 3 | 97594 |
| 5 | exp inflammatory bowel disease/ | 105586 |
| 6 | Guillain Barre syndrome/ | 11626 |
| 7 | irritable colon/ | 18690 |
| 8 | reactive arthritis/ | 2922 |
| 9 | hemolytic uremic syndrome/ | 8993 |
| 10 | (Sequel* or Complication* or Long-term or Longterm or chronic or Guillain* or HUS or Haemolytic uremic syndrome or Hemolytic uremic syndrome or Hemorrhagic uremic syndrome or Haemorrhagic uraemic syndrome or Reiter* or Arthritis* or Irritable bowel syndrome or IBS or inflammatory bowel disease or colitis or crohn*).ti,ab. | 3044735 |
| 11 | 5 or 6 or 7 or 8 or 9 or 10 | 3073733 |
| 12 | 4 and 11 | 7690 |
| 13 | limit 12 to yr="2011 -Current" | 2132 |

## CAB Abstracts

| **# ▲** | **Searches** | **Results** |
| --- | --- | --- |
| 1 | (salmonella* or campylobacter*).ti,ab. | 33356 |
| 2 | salmonella/ or salmonellosis/ | 30603 |
| 3 | exp campylobacter/ | 6653 |
| 4 | salmonella.od. | 18096 |
| 5 | campylobacter*.od. | 6626 |
| 6 | 1 or 2 or 3 or 4 or 5 | 37086 |
| 7 | exp inflammatory bowel diseases/ | 2975 |
| 8 | irritable colon.sh. | 1300 |
| 9 | arthritis.sh. | 4077 |
| 10 | Guillain-Barre syndrome.sh. | 138 |
| 11 | haemolytic uraemic syndrome.sh. | 298 |
| 12 | (Sequel* or Complication* or Long-term or Longterm or chronic or Guillain* or HUS or Haemolytic uremic syndrome or Hemolytic uremic syndrome or Hemorrhagic uremic syndrome or Haemorrhagic uraemic syndrome or Reiter* or Arthritis* or Irritable bowel syndrome or IBS or inflammatory bowel disease or colitis or crohn*).ti,ab. | 221963 |
| 13 | 7 or 8 or 9 or 10 or 11 or 12 | 222937 |
| 14 | 6 and 13 | 1032 |
| 15 | limit 14 to yr="2011 -Current" | 433 |

## Agricola

| **Query** | **Results** |
| --- | --- |
| (salmonella OR campylobacter) AND (guillain OR arthritis OR "inflammatory bowel" OR "irritable bowel" OR colitis OR crohns) | 32 |
| (salmonella OR campylobacter) AND (sequelae OR Complication OR complications OR long-term OR longterm OR chronic) | 0 |
| (salmonella OR campylobacter) AND "uremic syndrome" | 0 |

## Search summary

| **Database** | **Interface** | **Coverage** | **Dates** | **Hits** | **After duplicates** |
| --- | --- | --- | --- | --- | --- |
| AGRICOLA | <http://agricola.nal.usda.gov/> |  | 29.04/2016 | 32 | 32 |
| CAB abstracts | OvidSP | 2000 to 2016 Week 15 | 28/04/2016 | 433 | 182 |
| Embase | OvidSP | 1974 to 2016 April 27 | 28/04/2016 | 2132 | 1036 |
| PubMed | [http://www.pubmed.gov](http://www.pubmed.gov/) |  | 28/04/2016 | 1536 | 1536 |
| **Total:** |  |  |  | **4133** | **2786** |
| **Animal & plant studies excluded** |  |  |  | **363** |  |
| Duplicates: |  |  |  | 1347 |  |
| **Final total:** |  |  |  | **2422** |  |

# Supplementary Table S2. Data extraction tool

| **Variable** | **Description** |
| --- | --- |
| Entry ID | Unique ID for each entry on the data extraction form. First available Id is “1002” and automatically generated for the rest. |
| Article ID | Reference ID, See Article ID Worksheet for the list. |
| Author | Last name of first author. |
| Year of publication | Year article published |
| Country | Country from which study population is taken |
| Study design | The study design used to gather the information pertaining to the pathogen and chronic sequelae of interest. Options are:  - Retrospective outbreak*: used historic outbreak and historic disease status records.  - Prospective outbreak*: cases of pathogen identified from current or recent outbreak and followed forward in time to determine chronic sequelae status.  - Retrospective population surveillance: historic cases of pathogen and disease identified from population surveillance and connected.  - Prospective population surveillance: case of pathogen identified from population surveillance, followed forward in time to determine disease status.  - Cohort: study begins before illness from pathogen occurs. Cases and controls are identified and both are followed forward in time to determine pathogen exposure and associated sequelae status.  - Case control: cases of pathogen are identified, matched with controls and followed forward to time to determine disease status.  - Cross sectional: looks at population at a point in time. Not linked to outbreak or population surveillance data.  - Other  - Unknown  **Note: some studies will use a case control study to determine the source/risk factors of the outbreak. This would* ***NOT*** *be classified as a case control study as it does not pertain to the data on the pathogen/sequelae relationship.* |
| Source of data | What type of data source was used to gather the information used in the analysis. Options are:  - outbreak in hospital  - outbreak in community  - hospitalized cases  - surveillance of population (sporadic or outbreak) – this is meant to imply that ongoing population surveillance will often capture both sporadic and outbreak cases.  - disease registry  - notifiable diseases  - sporadic cases  - unknown  - other |
| Outbreak source | For outbreak data, indicate the source of the  outbreak. Options are:  - food - meat  - food - vegetable  - food- other  - food - dairy  - waterborne  - direct animal contact (e.g. farm/zoo/pets)  - daycare/nursery  - unknown  Otherwise enter NA (not applicable) |
| Date for data collection | Year (or range of years) data was collected. If not specifically reported enter “pre-publication date” (e.g. Pre-1994). Do not need to include days/months. If multiple years were included, separate with a comma (2004, 2010) unless data collection occurred over the entire period; indicate a range with a hyphen (2004-2010). |
| Season | Indicate season applicable to range for data collection.  Definitions:  Fall – September, October, November  Winter – December, January, February  Spring – March, April, May  Summer –June, July, August  Overlap between seasons (e.g. fall/winter) and yearlong are acceptable. For data collection that captures more than one year enter “various”. |
| Age range/categories | Age range (in years) used for the population with pathogen/sequelae of interest. No median age needed. Report category (e.g. Adults over 18) only if no range is given. |
| Gender | Indicate the gender mix of the population by reporting the number or (proportion if number not available) of participants who were female (“Gender F”) and who were male (“Gender M”). If information is not reported enter NR in each column. |
| Pathogen | Enter pathogen name. If there are multiple pathogens per study, create new rows following the first for each pathogen/sequelae combination. You will need to repeat the Article ID and Author in these cases.  Options:  - E. coli  - Campylobacter  - Salmonella |
| Diagnostic Test Used (Pathogen) | Specify the type of test used to diagnose the pathogen. The options are:  - Laboratory confirmed  - Probable cases based on case definition from study (for example Walkerton Health Study)  - Other  - Not reported  Classify on level most appropriate to outcome analysis.  Create new rows to report pathogen diagnosis for different outcomes.  *For example: if the study reports the number of cases of HUS associated with all “probable cases based on case definition from study” (e.g: 10 out of 300 probable cases – outcome 1 (10/300), row 1) as well as the number of cases of HUS associated with culture confirmed cases (of the 10 HUS overall, 9 were patients who were culture confirmed out of a total of 150 culture confirmed cases – outcome 2(9/150), row 2) this would be two separate rows with two separate pathogen diagnosis and outcomes for the same study. Can only do this when the paper SPECIFICALLY says that the chronic sequelae cases were also lab confirmed.* |
| Pathogen Subtype | Enter species name or serotype (salmonella) (NOT CAPITALIZED) (E.g.: jejuni NOT Jejuni). Enter NR (not reported) if it’s not specified. Create new rows for each species/serotype given ONLY IF outcome information is available at the species/serotype level.  *For example: A study looked at campylobacter and differentiated between C. jejuni and C. fetus and reported outcomes for both; The number of people positive for campylobacter jejuni who developed IBS was 1/100 (outcome 1, row 1), the number of people positive for campylobacter fetus who developed IBS was 0/130 (outcome 2, row 2). The total number of people with campylobacter who developed IBS was 1/230 (outcome 3, row 3).* |
| Fever | Record the number or proportion reported in the study |
| Vomiting | Record the number or proportion reported in the study |
| Diarrhoea | Record the number or proportion reported in the study |
| Bloody Diarrhoea | Record the number or proportion reported in the study |
| Abdominal pain | Record the number or proportion reported in the study |
| GP Visit/Physician | Record the number or proportion reported in the study |
| A&E/emergency Department | Record the number or proportion reported in the study |
| Hospitalised | Record the number or proportion reported in the study |
| Antibiotic Reported | Options:  - Yes  - No |
| Antibiotics Numbers | Record the number or proportion reported in the study. If the name of the antibiotics is given indicate this. E.g (Azithromycin – 3/10) |
| Other drugs | Record the number or proportion reported in the study. If the name of the drug is given indicate this. E.g. (Zantac – 3/10) |
| Underlying condition reported | Options  -Yes  -No |
| Underlying condition notes | Record the name of the condition and the number of people/proportion. E.g. cancer -3/10 |
| Complication | Specify which sequelae the study reports on as an outcome. If there are multiple sequelae per study, create new rows for each pathogen/sequelae combination. You will need to manually enter the ArticleID and author in these cases.  Sequelae of interest are:  - HUS (Haemolytic Anaemia, Thrombocytopenia)  - Renal Failure/Decreased Renal Function  - Reactive Arthritis (ReA)  - Reiters  - Combined ReA/Reiters  - Irritable Bowel Syndrome  - Post Infectious Irritable Bowel Syndrome (PI-IBS)  - Crohn's Disease  - Ulcerative Colitis  - Inflammatory Bowel Disease  - Guillain Barré (Acute idiopathic polyneuritis/Infectious polyneuritis  - Acute inflammatory polyneuropathy; Acute inflammatory demyelinating polyneuropathy)  - Miller-Fisher Syndrome |
| Diagnosis (Sequelae) | Specify how the diagnosis for the sequelae was performed. Options include:  - Self reported disease status (E.g. Participant says they have ReA)  - Self reported based on validated scale (E.g. Used Questionnaire Utilizing Epidemic Spondyloarthropathy Traits 2 (QUEST2) survey for ReA diagnosis)  - Disease status confirmed by specialist (diagnosis of ReA confirmed by Rheumatologist)  - Other trained health professional (diagnosis performed by trained health professionals. E.g. Nurses trained for research project)  - Not reported  - Combination (If more than one method was used, stated methods used in the comments section) |
| Follow up | Time from Illness from Pathogen to Evaluation for complication. Enter the maximum length of time (in days) between onset of illness and evaluation for the chronic sequelae. Enter NR if not reported. |
| Number of people with pathogen (denominator) | Number of people with the pathogen who are used for the analysis for the outcome of interest. |
| Number of people with sequelae (numerator) | Number of people with the pathogen who went on to develop the chronic sequelae of interest |
| Funding Source | Record what is reported, if blank, Enter NR |
| Conflicts of interest | Record what is reported, if blank, Enter NR |
| Comments | Insert any comments regarding the study design, symptoms or outcome |

# Supplementary Table S3. Risk of Bias Tool

| **Author & Year** | **Q1** | **Q2** | **Q3** | **Q4** | **Q5** | **Q6*** | **Q7** | **Q8*** | **Q9** | **Q10** | **Q11** | **Q12** |
| --- | --- | --- | --- | --- | --- | --- | --- | --- | --- | --- | --- | --- |
| (Arnedo-Pena et al., 2010) |  |  |  |  |  |  |  |  |  |  |  |  |
| (Baker et al., 2012) |  |  |  |  |  |  |  |  |  |  |  |  |
| (Bremell et al., 1991) |  |  |  |  |  |  |  |  |  |  |  |  |
| (Buxton et al., 2002) |  |  |  |  |  |  |  |  |  |  |  |  |
| (Doorduyn et al., 2008) |  |  |  |  |  |  |  |  |  |  |  |  |
| (Dunlop et al., 2003) |  |  |  |  |  |  |  |  |  |  |  |  |
| (Dworkin et al., 2001) |  |  |  |  |  |  |  |  |  |  |  |  |
| (Eastmond et al., 1981) |  |  |  |  |  |  |  |  |  |  |  |  |
| (Eastmond, 1983) |  |  |  |  |  |  |  |  |  |  |  |  |
| (Ekman et al., 2000) |  |  |  |  |  |  |  |  |  |  |  |  |
| (Gardner et al., 2011) |  |  |  |  |  |  |  |  |  |  |  |  |
| (Gumpel et al., 1981) |  |  |  |  |  |  |  |  |  |  |  |  |
| (Hakansson et al., 1976) |  |  |  |  |  |  |  |  |  |  |  |  |
| (Hannu et al., 2002a) |  |  |  |  |  |  |  |  |  |  |  |  |
| (Hannu et al., 2002b) |  |  |  |  |  |  |  |  |  |  |  |  |
| (Helms et al., 2006) |  |  |  |  |  |  |  |  |  |  |  |  |
| (Kosunen et al., 1981) |  |  |  |  |  |  |  |  |  |  |  |  |
| (Lee et al., 2005) |  |  |  |  |  |  |  |  |  |  |  |  |
| (Locht et al., 1993) |  |  |  |  |  |  |  |  |  |  |  |  |
| (Locht and Krogfelt, 2002) |  |  |  |  |  |  |  |  |  |  |  |  |
| (Locht et al., 2002) |  |  |  |  |  |  |  |  |  |  |  |  |
| (Mattila et al., 1994) |  |  |  |  |  |  |  |  |  |  |  |  |
| (Mattila et al., 1998) |  |  |  |  |  |  |  |  |  |  |  |  |
| (McCarthy et al., 1999) |  |  |  |  |  |  |  |  |  |  |  |  |
| (McCarthy and Giesecke, 2001) |  |  |  |  |  |  |  |  |  |  |  |  |
| (McColl et al., 2000) |  |  |  |  |  |  |  |  |  |  |  |  |
| (McKendrick and Read, 1994) |  |  |  |  |  |  |  |  |  |  |  |  |
| (Mearin et al., 2005) |  |  |  |  |  |  |  |  |  |  |  |  |
| (Melby et al., 1990) |  |  |  |  |  |  |  |  |  |  |  |  |
| (Moss-Morris and Spence, 2006) |  |  |  |  |  |  |  |  |  |  |  |  |
| (Petersen et al., 1996) |  |  |  |  |  |  |  |  |  |  |  |  |
| (Pitkanen et al., 1981) |  |  |  |  |  |  |  |  |  |  |  |  |
| (Pitkanen et al., 1983) |  |  |  |  |  |  |  |  |  |  |  |  |
| (Ponka et al., 1984) |  |  |  |  |  |  |  |  |  |  |  |  |
| (Porter et al., 2013b) |  |  |  |  |  |  |  |  |  |  |  |  |
| (Porter et al., 2013a) |  |  |  |  |  |  |  |  |  |  |  |  |
| (Rohekar et al., 2008) |  |  |  |  |  |  |  |  |  |  |  |  |
| (Rudwaleit et al., 2001) |  |  |  |  |  |  |  |  |  |  |  |  |
| (Samuel et al., 1995) |  |  |  |  |  |  |  |  |  |  |  |  |
| (Saps et al., 2008) |  |  |  |  |  |  |  |  |  |  |  |  |
| (Schiellerup et al., 2008) |  |  |  |  |  |  |  |  |  |  |  |  |
| (Schonberg-Norio et al., 2010) |  |  |  |  |  |  |  |  |  |  |  |  |
| (Short et al., 1982) |  |  |  |  |  |  |  |  |  |  |  |  |
| (Spence and Moss-Morris, 2007) |  |  |  |  |  |  |  |  |  |  |  |  |
| (Spiller et al., 2000) |  |  |  |  |  |  |  |  |  |  |  |  |
| (Tam et al., 2006) |  |  |  |  |  |  |  |  |  |  |  |  |
| (Ternhag et al., 2008) |  |  |  |  |  |  |  |  |  |  |  |  |
| (Thomson et al., 1992) |  |  |  |  |  |  |  |  |  |  |  |  |
| (Thomson et al., 1994) |  |  |  |  |  |  |  |  |  |  |  |  |
| (Thornley et al., 2001) |  |  |  |  |  |  |  |  |  |  |  |  |
| (Townes et al., 2008) |  |  |  |  |  |  |  |  |  |  |  |  |
| (Tuompo et al., 2013) |  |  |  |  |  |  |  |  |  |  |  |  |
| (Uotila et al., 2014) |  |  |  |  |  |  |  |  |  |  |  |  |
| (Urfer et al., 2000) |  |  |  |  |  |  |  |  |  |  |  |  |
| (Wang et al., 2008) |  |  |  |  |  |  |  |  |  |  |  |  |
| **Total number of studies with “Yes”** | 41 | 51 | 10 | 43 | 42 | 48 | 41 | 42 | 32 | 45 | 27 | 4 |

*The questions were repeated for the pathogen and sequelae as an adaptation to the tool.

|  | No |
| --- | --- |
|  | Unclear |
|  | Yes |

The risk of bias tool is an adapted version of the Joanna Briggs Critical Appraisal Tool. Total score per paper was not assigned. The full questions are listed below:

**Questions**

| **Q1. Was the sample frame appropriate to address the target population?** |
| --- |
| **Q2. Were study participants sampled in an appropriate way (use of relevant data sources)?** |
| **Q3. Was the sample size adequate?** |
| **Q4. Were the study subjects and the setting described?** |
| **Q5. Was the data analysis conducted with sufficient coverage of the identified sample?** |
| **Q6. Were valid methods used for the identification of the pathogen?** |
| **Q7. Were valid methods used for the identification of the complication?** |
| **Q8. Was the pathogen measured according to standard practice for all participants?** |
| **Q9. Was the complication measured according to standard practice for all participants?** |
| **Q10. Was there appropriate statistical analysis?** |
| **Q11. Was the response rate adequate?** |
| **Q12. If response rate was inadequate, was the low response rate managed appropriately?** |

# Supplementary Table S4A. Proportion developing complications following *Campylobacter* infection

| **Author & Year** | **Pathogen Subtype** | **Pathogen_Diagnosis** | **Complication** | **Diagnosis_ Complication** | **Definition of Complication** | **Follow-up** | **No. with pathogen** | **No. with complication** | **Proportion** |
| --- | --- | --- | --- | --- | --- | --- | --- | --- | --- |
| **Guillain Barré Syndrome** | | | | | | | | | |
| (Baker et al., 2012) | N.R | Laboratory Confirmed | N.R | Specialist | Yes - Validated Criteria | 60 | 8448 | 34 | 0.40% |
| (Doorduyn et al., 2008) | N.R | Laboratory Confirmed | N.R | Self-reported disease status | N.R | 1080 | 434 | 0 | 0.00% |
| (Gardner et al., 2011) | *C. jejuni* | Laboratory Confirmed | No | Specialist | N.R | 9 | 45 | 1 | 2.22% |
| (Helms et al., 2006) | N.R | Laboratory Confirmed | No | Physician/Medical records | N.R | 365 | 17991 | 6 | 0.03% |
| (McCarthy et al., 1999) | N.R | Probable | No | Physician/Medical records | Yes - Author's Definition | 180 | 8086 | 0 | 0.0% |
| (McCarthy and Giesecke, 2001) | *C. jejuni* | Laboratory Confirmed | No | Physician/Medical records | Yes - Validated Criteria | 180 | 29563 | 9 | 0.03% |
| (Tam et al., 2006) | N.R | Laboratory Confirmed | No | Physician/Medical records | Yes - Validated Criteria | 60 | 15587 | 3 | 0.02% |
| (Wang et al., 2008) | *C. coli* | Laboratory Confirmed | No | Physician/Medical records | N.R | N.R | 24 | 0 | 0.00% |
| (Wang et al., 2008) | *C. jejuni* | Laboratory Confirmed | No | Physician/Medical records | N.R | N.R | 80 | 0 | 0.00% |
| (Wang et al., 2008)* | *C. jejuni/coli* | Laboratory Confirmed | No | Physician/Medical records | N.R | N.R | 104 | 0 | 0.00% |
| **Irritable bowel syndrome** | | | | | | | | | |
| (Dunlop et al., 2003) | *C. coli/jejuni* | Laboratory Confirmed | No | Self-reported based on validated scale | Yes - Validated Criteria | 90 | 747 | 103 | 13.79% |
| (Helms et al., 2006) | N.R | Laboratory Confirmed | No | Physician/Medical records | N.R | 365 | 17991 | 161 | 0.89% |
| (Moss-Morris and Spence, 2006) | N.R | Laboratory Confirmed | No | Self-reported based on validated scale | N.R | 90 | 553 | 83 | 15.01% |
| (Moss-Morris and Spence, 2006) | N.R | Laboratory Confirmed | No | Self-reported based on validated scale | N.R | 180 | 536 | 59 | 11.01% |
| (Porter et al., 2013a) | N.R | Laboratory Confirmed | No | Physician/Medical records | Yes - Validated Criteria | N.R | 738 | 37 | 5.01% |
| (Saps et al., 2008) | N.R | Laboratory Confirmed | N.R | Self-reported based on validated scale | Yes -Validated Criteria | 180 | 6 | 1 | 16.67% |
| (Spence and Moss-Morris, 2007) | *C. jejuni* | Laboratory Confirmed | No | Self-reported based on validated scale | N.R | 180 | 620 | 49 | 7.90% |
| (Spiller et al., 2000) | *C. jejuni* | Laboratory Confirmed | N.R | Self-reported based on validated scale | N.R | 365 | 31 | 4 | 12.90% |
| (Ternhag et al., 2008) | *C. coli/jejuni* | Laboratory Confirmed | NR | Physician/Medical records | N.R | 365 | 57425 | 15 | 0.03% |
| (Thornley et al., 2001) | N.R | Laboratory Confirmed | No | Self-reported based on validated scale | N.R | 180 | 188 | 17 | 9.04% |
| **Reactive arthritis** | | | | | | | | | |
| (Bremell et al., 1991) | *C. jejuni* | Laboratory Confirmed | N.R | Self-reported disease status | Yes - Author's Definition | 30 | 66 | 1 | 1.52% |
| (Doorduyn et al., 2008) | N.R | Laboratory Confirmed | N.R | Self-reported disease status | N.R | 1080 | 434 | 20 | 4.61% |
| (Eastmond et al., 1981) | N.R | Laboratory Confirmed | No | Physician/Medical records | N.R | 90 | 88 | 1 | 1.14% |
| (Gumpel et al., 1981) | N.R | Laboratory Confirmed | N.R | Physician/Medical records | N.R | N.R | 77 | 8 | 10.39% |
| (Hannu et al., 2002a)* | *C. coli/jejuni/undetermined* | Laboratory Confirmed | No | Specialist | Yes - Author's Definition | 60 | 609 | 45 | 7.39% |
| (Hannu et al., 2002a) | *C. coli/jejuni/undetermined* | Laboratory Confirmed | No | Self-reported disease status | Yes - Author's Definition | 60 | 609 | 7 | 1.15% |
| (Hannu et al., 2002a) | *C. jejuni* | Laboratory Confirmed | No | Specialist | Yes - Author's Definition | 60 | 535 | 37 | 6.92% |
| (Hannu et al., 2002a) | *C. coli* | Laboratory Confirmed | No | Specialist | Yes - Author's Definition | 60 | 61 | 8 | 13.11% |
| (Hannu et al., 2002a) | Undetermined | Laboratory Confirmed | No | Specialist | Yes - Author's Definition | 60 | 13 | 0 | 0.00% |
| (Helms et al., 2006) | N.R | Laboratory Confirmed | No | Physician/Medical records | N.R | 365 | 17991 | 22 | 0.12% |
| (Kosunen et al., 1981) | *C. jejuni* | Laboratory Confirmed | N.R | Not reported | N.R | 365 | 342 | 8 | 2.34% |
| 2002(Locht and Krogfelt, 2002) | *C. coli/jejuni* | Laboratory Confirmed | No | Self-reported disease status | Yes - Author's Definition | 28 | 173 | 27 | 15.61% |
| (Petersen et al., 1996) | *C. coli/jejuni* | Laboratory Confirmed | N.R | Physician/Medical records | N.R | N.R | 41 | 0 | 0.00% |
| (Pitkanen et al., 1983) | *C. jejuni* | Laboratory Confirmed |  | Combination | N.R | N.R | 188 | 9 | 4.79% |
| (Pitkanen et al., 1981) | *C. jejuni* | Laboratory Confirmed | N.R | Combination | N.R | N.R | 56 | 4 | 7.14% |
| (Ponka et al., 1984) | *C. jejuni* | Laboratory Confirmed | N.R | Self-reported disease status | N.R | N.R | 383 | 6 | 1.57% |
| (Schiellerup et al., 2008) | N.R | Laboratory Confirmed | No | Self-reported based on validated scale | Yes -Validated Criteria | 28 | 1003 | 131 | 13.06% |
| (Schonberg-Norio et al., 2010) | *C. jejuni* | Laboratory Confirmed | No | Physician/Medical records | Yes - Author's Definition | 60 | 201 | 8 | 3.98% |
| (Short et al., 1982) | *C. jejuni* | Laboratory Confirmed | N.R | Physician/Medical records | N.R | 42 | 15 | 0 | 0.00% |
| (Ternhag et al., 2008)* | *C.coli/jejuni* | Laboratory Confirmed | N.R | Physician/Medical records | N.R | 365 | 57425 | 15 | 0.03% |
| (Ternhag et al., 2008) | *C.coli/jejuni* | Laboratory Confirmed | N.R | Physician/Medical records | N.R | 90 | 57425 | 13 | 0.02% |
| (Townes et al., 2008) | N.R | Laboratory Confirmed | No | Physician/Medical records | Yes - Author's Definition | 56 | 2384 | 33 | 1.38% |
| (Uotila et al., 2014) | N.R | Laboratory Confirmed | No | Self-reported disease status | Yes - Author's definition | 630 | 73 | 42 | 57.53% |
| **Reiters syndrome** | | | | | | | | | |
| (Doorduyn et al., 2008) | N.R | Laboratory Confirmed | No | Self-reported disease status | N.R | 1080 | 434 | 0 | 0.00% |
| (Porter et al., 2013b) | N.R | Laboratory Confirmed | No | Physician/Medical records | Yes - Validated Criteria | 180 | 738 | 1 | 0.14% |

Yes - Validated Criteria^‡^ - These include ICD-9/ICD-10 definition, validated questionnaire definition such as Rome I, Rome II, Rome III criteria for IBS

# Supplementary Table S4B. Proportion developing complications following NTS infection

| **Author & Year** | **Pathogen Serotype^†^** | **Pathogen_Diagnosis** | **Complication** | **Diagnosis_ Complication** | **Definition of Complication^‡^** | **Follow-up** | **No. with pathogen** | **No. with complication** | **Proportion** |
| --- | --- | --- | --- | --- | --- | --- | --- | --- | --- |
| **Irritable bowel syndrome** | | | | | | | | | |
| (Helms et al., 2006)* | Multiple | Laboratory Confirmed | No | Physician/Medical records | N.R | 365 | 27894 | 252 | 0.90% |
| (Helms et al., 2006) | Enteritidis | Laboratory Confirmed | No | Physician/Medical records | N.R | 365 | 14533 | 125 | 0.86% |
| (Helms et al., 2006) | Typhimurium | Laboratory Confirmed | No | Physician/Medical records | N.R | 365 | 7021 | 67 | 0.95% |
| (Helms et al., 2006) | Other NTS | Laboratory Confirmed | No | Physician/Medical records | N.R | 365 | 6034 | 60 | 0.99% |
| McKendrick 1994(McKendrick and Read, 1994) | Enteritidis PT4 | Probable | No | Self-reported based on validated scale | Yes - Validated Criteria | 365 | 38 | 12 | 31.58% |
| (Mearin et al., 2005) | Enteritidis | Probable | N.R | Self-reported based on validated scale | Yes - Validated Criteria | 365 | 266 | 31 | 11.65% |
| (Porter et al., 2013a) | N.R | Laboratory Confirmed | No | Physician/Medical records | Yes - Validated Criteria | NR | 624 | 32 | 5.13% |
| (Saps et al., 2008) | N.R | Laboratory Confirmed | N.R | Self-reported based on validated scale | Yes -Validated Criteria | 180 | 24 | 9 | 37.50% |
| (Ternhag et al., 2008) | Multiple | Laboratory Confirmed | N.R | Physician/Medical records | N.R | 365 | 34664 | 5 | 0.01% |
| (Urfer et al., 2000) | Braenderup | Laboratory Confirmed | N.R | Self-reported | N.R | 180 | 127 | 12 | 9.45% |
| **Reactive arthritis** | | | | | | | | | |
| (Arnedo-Pena et al., 2010) | Hadar | Probable | No | Self-reported based on validated scale | Yes - Author's Definition | 90 | 155 | 16 | 10.32% |
| (Arnedo-Pena et al., 2010)* | Hadar PT2 | Laboratory Confirmed | No | Specialist | Yes - Author's Definition | 90 | 67 | 6 | 8.96% |
| (Arnedo-Pena et al., 2010) | Hadar | Probable | No | Specialist | Yes - Author's Definition | 90 | 155 | 13 | 8.39% |
| Buxton 2002(Buxton et al., 2002) | Typhimurium | Laboratory Confirmed | No | Self-reported based on validated scale | Yes - Validated Criteria | 120 | 66 | 17 | 25.76% |
| (Buxton et al., 2002) | Typhimurium | Laboratory Confirmed | No | Specialist | Yes - Author's Definition | 90 | 61 | 4 | 6.56% |
| (Doorduyn et al., 2008) | N.R | Laboratory Confirmed | Yes | Self-reported | N.R | 1080 | 181 | 8 | 4.42% |
| Dworkin 2001 | Enteritidis | Probable | N.R | Self-reported | Yes - Author's definition | 30 | 217 | 63 | 29.03% |
| (Eastmond, 1983) | N.R | Laboratory Confirmed | N.R | Physician/Medical records | Yes - Author's definition | 60 | 418 | 8 | 1.91% |
| (Ekman et al., 2000)* | N.R | Laboratory Confirmed | No | Specialist | N.R | N.R | 198 | 8 | 4.04% |
| (Ekman et al., 2000) | N.R | Laboratory Confirmed | No | Self-reported | N.R | N.R | 198 | 13 | 6.57% |
| (Hakansson et al., 1976) | Typhimurium | Laboratory Confirmed | N.R | N.R | N.R | N.R | 330 | 13 | 3.94% |
| (Hannu et al., 2002b) | Typhimurium DT 193 | Laboratory Confirmed | No | Specialist | Yes - Author's definition | 60 | 63 | 5 | 7.94% |
| (Helms et al., 2006)* | Multiple | Laboratory Confirmed | No | Physician/Medical records | N.R | 365 | 27894 | 87 | 0.31% |
| (Helms et al., 2006) | Enteritidis | Laboratory Confirmed | No | Physician/Medical records | N.R | 365 | 14533 | 50 | 0.34% |
| (Helms et al., 2006) | Typhimurium | Laboratory Confirmed | No | Physician/Medical records | N.R | 365 | 7021 | 24 | 0.34% |
| (Helms et al., 2006) | other NTS | Laboratory Confirmed | No | Physician/Medical records | N.R | 365 | 6034 | 13 | 0.22% |
| (Lee et al., 2005)* | Typhimurium PT 135a | Laboratory Confirmed | No | Physician/Medical records | Yes - Author's definition | 90 | 261 | 38 | 14.56% - All |
| (Lee et al., 2005) | Typhimurium PT 135a | Laboratory Confirmed | No | Physician/Medical records | Yes - Author's definition | 90 | 54 | 13 | 24.07% - Children |
| (Lee et al., 2005) | Typhimurium PT 135a | Laboratory Confirmed | No | Physician/Medical records | Yes - Author's definition | 90 | 207 | 25 | 12.08% - Adults |
| (Locht et al., 1993) | Enteritidis | Probable | N.R | Self-reported | Yes - Author's Definition | 28 | 108 | 17 | 15.74% |
| (Locht et al., 1993)* | Enteritidis | Laboratory Confirmed | N.R | Self-reported | Yes - Author's Definition | 28 | 89 | 16 | 17.98% |
| (Locht et al., 2002) | N.R | Probable | No | Self-reported | Yes - Author's definition | 28 | 91 | 17 | 18.68% |
| (Mattila et al., 1994) | Monophasic | Laboratory Confirmed | N.R | Specialist | Yes - Author's definition | 150 | 246 | 16 | 6.50% |
| (Mattila et al., 1998) | Bovismorbificans | Laboratory Confirmed | No | Specialist | Yes - Author's definition | 90 | 191 | 22 | 11.52% |
| (McColl et al., 2000)* | Tyhimurium | Probable | N.R | Self-reported | Yes - Author's definition | 90 | 312 | 13 | 4.17% |
| (McColl et al., 2000) | Tyhimurium PT9 | Probable | N.R | Self-reported | Yes - Author's definition | 90 | 112 | 6 | 5.36% |
| (Petersen et al., 1996) | Multiple | Laboratory Confirmed | N.R | Physician/Medical records | N.R | N.R | 127 | 7 | 5.51% |
| (Petersen et al., 1996) | Tyhimurium | Laboratory Confirmed | N.R | Physician/Medical records | N.R | N.R | 40 | 3 | 7.50% |
| (Petersen et al., 1996) | Enteritidis | Laboratory Confirmed | N.R | Physician/Medical records | N.R | N.R | 48 | 4 | 8.33% |
| (Petersen et al., 1996) | Other NTS | Laboratory Confirmed | N.R | Physician/Medical records | N.R | N.R | 39 | 0 | 0.00% |
| (Rohekar et al., 2008) | Enteritidis PT 13 | Laboratory Confirmed | N.R | Self-reported | Yes - Validated Criteria | N.R | 104 | 65 | 62.50% |
| (Rudwaleit et al., 2001) | Enteritidis | Laboratory Confirmed | No | Physician/Medical records | N.R | 120 | 286 | 0 | 0.00% |
| (Samuel et al., 1995) | N.R | Probable | N.R | Physician/Medical records | N.R | 112 | 321 | 23 | 7.17% |
| (Schiellerup et al., 2008)* | Multiple | Laboratory Confirmed | No | Self-reported based on validated scale | Yes -Validated Criteria | 28 | 619 | 104 | 16.80% |
| (Schiellerup et al., 2008) | Typhimurium | Laboratory Confirmed | No | Self-reported based on validated scale | Yes -Validated Criteria | 28 | 193 | 29 | 15.03% |
| (Schiellerup et al., 2008) | Enteritidis | Laboratory Confirmed | No | Self-reported based on validated scale | Yes -Validated Criteria | 28 | 270 | 49 | 18.15% |
| (Schiellerup et al., 2008) | Other NTS | Laboratory Confirmed | No | Self-reported based on validated scale | Yes -Validated Criteria | 28 | 156 | 26 | 16.67% |
| (Ternhag et al., 2008) | Multiple | Laboratory Confirmed | N.R | Physician/Medical records | N.R | 365 | 34664 | 27 | 0.08% |
| (Thomson et al., 1994) | Enteritidis | Probable | No | Physician/Medical records | Yes - Author's Definition | 60 | 29 | 8 | 27.59% |
| (Thomson et al., 1992) | Multiple | Probable | No | Self-reported | Yes - Author's definition | 28 | 73 | 6 | 8.22% |
| (Townes et al., 2008) | N.R | Laboratory Confirmed | No | Physician/Medical records | N.R | 56 | 1356 | 17 | 1.25% |
| (Tuompo et al., 2013) | Multiple | Laboratory Confirmed | No | Combination | Yes - Author's definition | 540 | 496 | 22 | 4.44% |
| (Urfer et al., 2000) | Braedenrup | Laboratory Confirmed | N.R | Self-reported | N.R | 180 | 127 | 1 | 0.79% |
| **Reiters syndrome** | | | | | | | | | |
| (Doorduyn et al., 2008) | N.R | Laboratory Confirmed | Yes | Self-reported | N.R | 1080 | 193 | 0 | 0.00% |
| (Dworkin et al., 2001) | Enteritidis | Probable | N.R | Self-reported | Yes - Author's definition | 30 | 217 | 6 | 2.76% |
| (Mattila et al., 1998) | Bovismorbificans | Laboratory Confirmed | No | Specialist | Yes - Author's definition | 90 | 191 | 0 | 0.00% |
| (Porter et al., 2013b) | N.R | Laboratory Confirmed | No | Physician/Medical records | Yes - Validated Criteria | 180 | 624 | 3 | 0.48% |
| (Thomson et al., 1994) | Enteritidis | Probable | No | Physician/Medical records | Yes - Author's Definition | 60 | 29 | 2 | 6.90% |

Author Year* - Outcome measures used for forest plots; Multiple^†^ - Enteritidis, Typhimurium & Other NTS serotypes

Yes - Validated Criteria^‡^ - These include ICD-9/ICD-10 definition, validated questionnaire definition such as Rome I, Rome II, Rome III criteria for IBS and QUEST2 - Questionnaire Utilizing Epidemic Spondyloarthropathy traits for reactive arthritis

**REFERENCES**

Arnedo-Pena, A., Beltran-Fabregat, J., Vila-Pastor, B., Tirado-Balaguer, M.D., Herrero-Carot, C., Bellido-Blasco, J.B., Romeu-Garcia, M.A., Safont-Adsuara, L., Pac-Sa, M.R., and Guillen-Grima, F. (2010). Reactive arthritis and other musculoskeletal sequelae following an outbreak of Salmonella hadar in Castellon, Spain. J Rheumatol *37*, 1735-1742.

Baker, M.G., Kvalsvig, A., Zhang, J., Lake, R., Sears, A., and Wilson, N. (2012). Declining Guillain-Barre syndrome after campylobacteriosis control, New Zealand, 1988-2010. Emerging infectious diseases *18*, 226-233.

Bremell, T., Bjelle, A., and Svedhem, A. (1991). Rheumatic symptoms following an outbreak of Campylobacter enteritis: A five year follow up. Ann Rheum Dis *50*, 934 - 938.

Buxton, J.A., Fyfe, M., Berger, S., Cox, M.B., and Northcott, K.A. (2002). Reactive arthritis and other sequelae following sporadic Salmonella typhimurium infection in British Columbia, Canada: a case control study. J Rheumatol *29*, 2154-2158.

Doorduyn, Y., Van Pelt, W., Siezen, C.L., Van Der Horst, F., Van Duynhoven, Y.T., Hoebee, B., and Janssen, R. (2008). Novel insight in the association between salmonellosis or campylobacteriosis and chronic illness, and the role of host genetics in susceptibility to these diseases. Epidemiol Infect *136*, 1225-1234.

Dunlop, S., Jenkins, D., Neal, K., and Spiller, R. (2003). Relative importance of enterochromaffin cell hyperplasia, anxiety, and depression in post infectious IBS. Gastroenterology *125*, 1651 - 1659.

Dworkin, M.S., Shoemaker, P.C., Goldoft, M.J., and Kobayashi, J.M. (2001). Reactive arthritis and Reiter's syndrome following an outbreak of gastroenteritis caused by Salmonella enteritidis. Clin Infect Dis *33*, 1010-1014.

Eastmond, C.J. (1983). Gram-negative bacteria and B27 disease. Br J Rheumatol *22*, 67-74.

Eastmond, C.J., Reid, T.M., and Rennie, J.A. (1981). Reactive arthritis associated with campylobacter enteritis. Ann Rheum Dis *40*, 431.

Ekman, P., Kirveskari, J., and Granfors, K. (2000). Modification of disease outcome in Salmonella-infected patients by HLA-B27. Arthritis Rheum *43*, 1527-1534.

Gardner, T., Fitzgerald, C., Xavier, C., Klein, R., Pruckler, J., Stroika, S., and McLaughlin, J. (2011). Outbreak of campylobacteriosis associated with consumption of raw peas. Clin Infect Dis *53*, 26 - 32.

Gumpel, J., Martin, C., and Sanderson, P. (1981). Reactive arthritis associated with Campylobacter enteritis. Ann Rheum Dis *40*, 64 - 65.

Hakansson, U., Eitrem, R., Low, B., and Winblad, S. (1976). HLA-antigen b27 in cases with joint affections in an outbreak of salmonellosis. Scandinavian journal of infectious diseases *8*, 245-248.

Hannu, T., Mattila, L., Rautelin, H., Pelkonen, P., Lahdenne, P., Siitonen, A., and Leirisalo-Repo, M. (2002a). Campylobacter-triggered reactive arthritis: A population-based study. Rheumatology *41*, 312 - 318.

Hannu, T., Mattila, L., Siitonen, A., and Leirisalo-Repo, M. (2002b). Reactive arthritis following an outbreak of Salmonella typhimurium phage type 193 infection. Ann Rheum Dis *61*, 264-266.

Helms, M., Simonsen, J., and Molbak, K. (2006). Foodborne bacterial infection and hospitalization: a registry-based study. Clin Infect Dis *42*, 498-506.

Kosunen, T., Ponka, A., Kauranen, O., Martio, J., Pitkanen, T., Hortling, L., Aittoniemi, S., Penttila, O., and Koskimies, S. (1981). Arthritis associated with Campylobacter jejuni/enteritis. Scand J Rheumatol *10*, 77 - 80.

Lee, A.T., Hall, R.G., and Pile, K.D. (2005). Reactive joint symptoms following an outbreak of Salmonella typhimurium phage type 135a. J Rheumatol *32*, 524-527.

Locht, H., Kihlstrom, E., and Lindstrom, F.D. (1993). Reactive arthritis after Salmonella among medical doctors--study of an outbreak. J Rheumatol *20*, 845-848.

Locht, H., and Krogfelt, K. (2002). Comparison of rheumatological and gastrointestinal symptoms after infection with Campylobacter jejuni/coli and enterotoxigenic Escherichia coli. Ann Rheum Dis *61*, 448 - 452.

Locht, H., Molbak, K., and Krogfelt, K.A. (2002). High frequency of reactive joint symptoms after an outbreak of Salmonella enteritidis. J Rheumatol *29*, 767-771.

Mattila, L., Leirisalo-Repo, M., Koskimies, S., Granfors, K., and Siitonen, A. (1994). Reactive arthritis following an outbreak of Salmonella infection in Finland. Br J Rheumatol *33*, 1136-1141.

Mattila, L., Leirisalo-Repo, M., Pelkonen, P., Koskimies, S., Granfors, K., and Siitonen, A. (1998). Reactive arthritis following an outbreak of Salmonella Bovismorbificans infection. J Infect *36*, 289-295.

McCarthy, N., Andersson, Y., Jormanainen, V., Gustavsson, O., and Giesecke, J. (1999). The risk of Guillain-Barre syndrome following infection with Campylobacter jejuni. Epidemiol Infect *122*, 15 - 17.

McCarthy, N., and Giesecke, J. (2001). Incidence of Guillain-Barre syndrome following infection with Campylobacter jejuni. Am J Epidemiol *153*, 610 - 614.

McColl, G.J., Diviney, M.B., Holdsworth, R.F., McNair, P.D., Carnie, J., Hart, W., and McCluskey, J. (2000). HLA-B27 expression and reactive arthritis susceptibility in two patient cohorts infected with Salmonella Typhimurium. Aust N Z J Med *30*, 28-32.

McKendrick, M.W., and Read, N.W. (1994). Irritable bowel syndrome--post salmonella infection. J Infect *29*, 1-3.

Mearin, F., Perez-Oliveras, M., Perello, A., Vinyet, J., Ibanez, A., Coderch, J., and Perona, M. (2005). Dyspepsia and irritable bowel syndrome after a Salmonella gastroenteritis outbreak: one-year follow-up cohort study. Gastroenterology *129*, 98-104.

Melby, K., Dahl, O., Crisp, L., and Penner, J. (1990). Clinical and serological manifestations in patients during a waterborne epidemic due to Campylobacter jejuni. J Infect *21*, 309 - 316.

Moss-Morris, R., and Spence, M. (2006). To "lump" or to "split" the functional somatic syndromes: Can infectious and emotional risk factors differentiate between the onset of chronic fatigue syndrome and irritable bowel syndrome? Psychosom Med *68*, 463 - 469.

Petersen, A.M., Nielsen, S.V., Meyer, D., Ganer, P., and Ladefoged, K. (1996). Bacterial gastroenteritis among hospitalized patients in a Danish County, 1991-93. Scand J Gastroenterol *31*, 906-911.

Pitkanen, T., Pettersson, T., Ponka, A., and Kosunen, T. (1981). Clinical and serological studies in patients with Campylobacter fetus/ssp/jejuni infection: clinical findings. Infection *9*, 274 - 278.

Pitkanen, T., Ponka, A., Peterson, T., and Kosunen, T. (1983). Campylobacter enteritis in 188 hospitalized patients. Arch Intern Med *143*, 215 - 219.

Ponka, A., Pitkanen, T., Sarna, S., and Kosunen, T. (1984). Infection due to Campylobacter jejuni: A report of 524 outpatients. Infection *12*, 175 - 178.

Porter, C.K., Choi, D., Cash, B., Pimentel, M., Murray, J., May, L., and Riddle, M.S. (2013a). Pathogen-specific risk of chronic gastrointestinal disorders following bacterial causes of foodborne illness. BMC gastroenterology *13*, 46.

Porter, C.K., Choi, D., and Riddle, M.S. (2013b). Pathogen-specific risk of reactive arthritis from bacterial causes of foodborne illness. The Journal of rheumatology *40*, 712-714.

Rohekar, S., Tsui, F.W., Tsui, H.W., Xi, N., Riarh, R., Bilotta, R., and Inman, R.D. (2008). Symptomatic acute reactive arthritis after an outbreak of salmonella. J Rheumatol *35*, 1599-1602.

Rudwaleit, M., Richter, S., Braun, J., and Sieper, J. (2001). Low incidence of reactive arthritis in children following a salmonella outbreak. Ann Rheum Dis *60*, 1055-1057.

Samuel, M.P., Zwillich, S.H., Thomson, G.T., Alfa, M., Orr, K.B., Brittain, D.C., Miller, J.R., and Phillips, P.E. (1995). Fast food arthritis--a clinico-pathologic study of post-Salmonella reactive arthritis. J Rheumatol *22*, 1947-1952.

Saps, M., Pensabene, L., Di Martino, L., Staiano, A., Wechsler, J., Zheng, X., and Di Lorenzo, C. (2008). Post-infectious functional gastrointestinal disorders in children. J Pediatr *152*, 812-816, 816 e811.

Schiellerup, P., Krogfelt, K.A., and Locht, H. (2008). A comparison of self-reported joint symptoms following infection with different enteric pathogens: effect of HLA-B27. J Rheumatol *35*, 480-487.

Schonberg-Norio, D., Mattila, L., Lauhio, A., Katila, M.L., Kaukoranta, S.S., Koskela, M., Pajarre, S., Uksila, J., Eerola, E., Sarna, S.*, et al.* (2010). Patient-reported complications associated with Campylobacter jejuni infection. Epidemiol Infect *138*, 1004-1011.

Short, C., Klouda, P., and Smith, L. (1982). Campylobacter jejuni/enteritis and reactive arthritis. Ann Rheum Dis *41*, 287 - 288.

Spence, M., and Moss-Morris, R. (2007). The cognitive behavioral model of irritable bowel syndrome: A prospective investigation of patients with gastroenteritis. Gut *56*, 1066 - 1071.

Spiller, R., Jenkins, D., Thornley, J., Hebden, J., Wright, T., Skinner, M., and Neal, K. (2000). Increased rectal mucosal enteroendocrine cells, T lymphocytes, and increased gut permeability following acute Campylobacter enteritis and in post-dysenteric irritable bowel syndrome. Gut *47*, 804 - 811.

Tam, C., Rodrigues, L., Petersen, I., Islam, A., Hayward, A., and O'Brien, S. (2006). Incidence of Guillain-Barre syndrome among patients with Campylobacter infection: A general practice research database study. J Infect Dis *194*, 95 - 97.

Ternhag, A., Torner, A., Svensson, A., Ekdahl, K., and Giesecke, J. (2008). Short- and long-term effects of bacterial gastrointestinal infections. Emerg Infect Dis *14*, 143-148.

Thomson, G.T., Alfa, M., Orr, K., Thomson, B.R., and Olson, N. (1994). Secretory immune response and clinical sequelae of Salmonella infection in a point source cohort. J Rheumatol *21*, 132-137.

Thomson, G.T., Chiu, B., De Rubeis, D., Falk, J., and Inman, R.D. (1992). Immunoepidemiology of post-Salmonella reactive arthritis in a cohort of women. Clin Immunol Immunopathol *64*, 227-232.

Thornley, J., Jenkins, D., Neal, K., Wright, T., Brough, J., and Spiller, R. (2001). Relationship of Campylobacter toxigenicity in vitro to the development of post infectious irritable bowel syndrome. J Infect Dis *184*, 606 - 609.

Townes, J.M., Deodhar, A.A., Laine, E.S., Smith, K., Krug, H.E., Barkhuizen, A., Thompson, M.E., Cieslak, P.R., and Sobel, J. (2008). Reactive arthritis following culture-confirmed infections with bacterial enteric pathogens in Minnesota and Oregon: a population-based study. Ann Rheum Dis *67*, 1689-1696.

Tuompo, R., Hannu, T., Mattila, L., Siitonen, A., and Leirisalo-Repo, M. (2013). Reactive arthritis following Salmonella infection: a population-based study. Scandinavian journal of rheumatology *42*, 196-202.

Uotila, T., Korpela, M., Vuento, R., Laine, J., Lumio, J., Kuusi, M., Virtanen, M.J., Mustonen, J., and Antonen, J. (2014). Joint symptoms after a faecal culture positive Campylobacter infection associated with a waterborne gastroenteritis outbreak: a questionnaire study. Scandinavian journal of rheumatology *43*, 524-526.

Urfer, E., Rossier, P., Mean, F., Krending, M.J., Burnens, A., Bille, J., Francioli, P., and Zwahlen, A. (2000). Outbreak of Salmonella braenderup gastroenteritis due to contaminated meat pies: clinical and molecular epidemiology. Clin Microbiol Infect *6*, 536-542.

Wang, S., Chang, L., Hsueh, P., Lu, C., Lee, P., Shao, P., Hsieh, Y., Yen, F., Lee, C., and Huang, L. (2008). Campylobacter enteritis in children in northern Taiwan-a 7-year experience. J Microbiol Immunol Infect *41*, 408 - 413.
